# Early ADME and Preliminary Toxicity Studies of I‑152, an *N*‑Acetyl‑l‑Cysteine/*S*‑Acetylcysteamine Conjugate

**DOI:** 10.1021/acs.chemrestox.6c00018

**Published:** 2026-04-27

**Authors:** Francesca Bartoccini, Aurora Valeri, Matteo Gregori, Sofia Masini, Michela Bruschi, Laura Goracci, Alessandra Fraternale, Giovanni Piersanti

**Affiliations:** † Department of Biomolecular Sciences, 19044 University of Urbino Carlo Bo, Via Ca’ Le Suore 2, Urbino 61029, Italy; ‡ DAISY Lab (Drug Discovery-Artificial Intelligence-Organic Synthesis), Department of Chemistry, Biology and Biotechnology, 9309 University of Perugia, Via Elce di Sotto 8, Perugia 06123, Italy; § Molecular Horizon srl, Via Montelino 30, Bettona 06084, Italy

## Abstract

This study aimed to estimate the ADME properties and safety of I-152, a conjugate of *N*-acetyl-l-cysteine (NAC) and *S*-acetylcysteamine (also known as *S*-acetyl-β-mercaptoethylamine; SMEA), linked by an amide bond. Its potent antioxidant and pro-glutathione effects make it of interest for a range of conditions linked to oxidative stress, such as infectious diseases and inflammation. I-152 was characterized in vitro for its stability in plasma, liver microsomes, and hepatocytes; its protein binding; and its AB BA (apical-to-basolateral and basolateral-to-apical) permeability using Caco-2 cells. Derisking and preliminary safety pharmacology assays were performed through a human ether-à-go-go-related gene assay (hERG) and in vitro cellular toxicity tests. The results demonstrated that I-152 is hydrolyzed in human plasma (half-life of about 9 min), human liver microsomes, and hepatocytes, as well as in rat liver microsomes and hepatocytes. In addition, I-152 was found to be permeable across the Caco-2 monolayer, indicating good intestinal absorption. Furthermore, I-152 did not produce detectable toxic effects at concentrations up to 1 mM in vitro assays using human keratinocytes, alveolar epithelial cells, and immortalized human embryonic kidney cells (HEK293T). These findings support further preclinical evaluation as a potential redox-modulating agent and thiol-based approach for viral infections and other conditions associated with oxidative stress.

## Graphical abstract

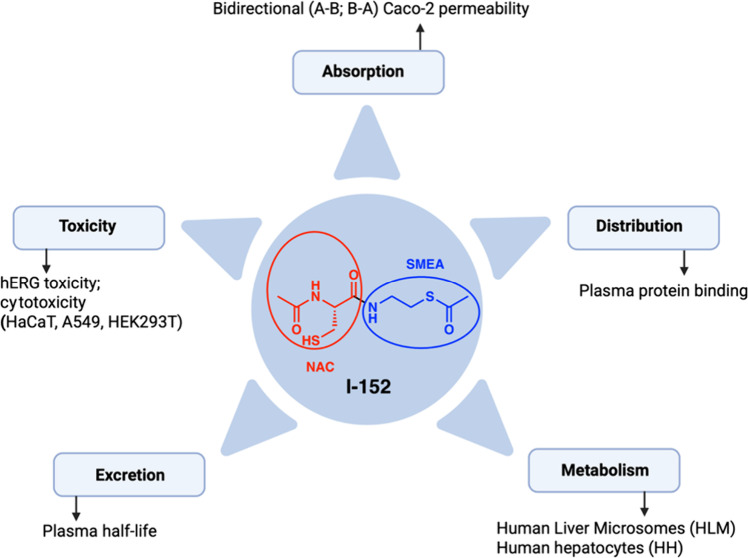

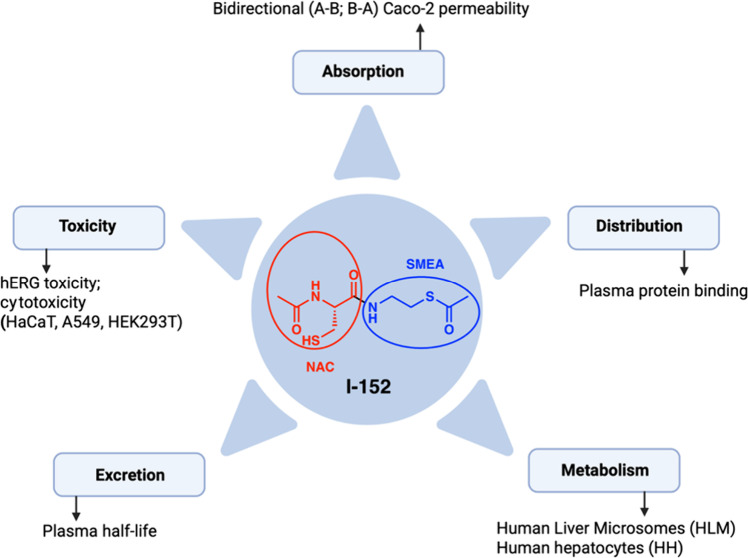

## 1 Introduction

I-152 is a synthetic compound containing *N*-acetyl-l-cysteine (NAC) and *S*-acetyl-cysteamine (also known as *S*-acetyl-β-mercaptoethylamine; SMEA), linked by an amide bond. It is prepared from *N*-acetylated cysteine and *S*-acyl cysteamine under standard amide coupling conditions (Figure 1).^1,2^ It was devised to combine these two sulfur-containing compounds into a single entity, since cysteine and cysteamine present several clinical applications but have limited efficacy due to undesirable properties (e.g., bitter taste, unpleasant odor, chemical instability, hygroscopicity, etc.) and a poor pharmacokinetic profile.^3^

**Figure 1:**
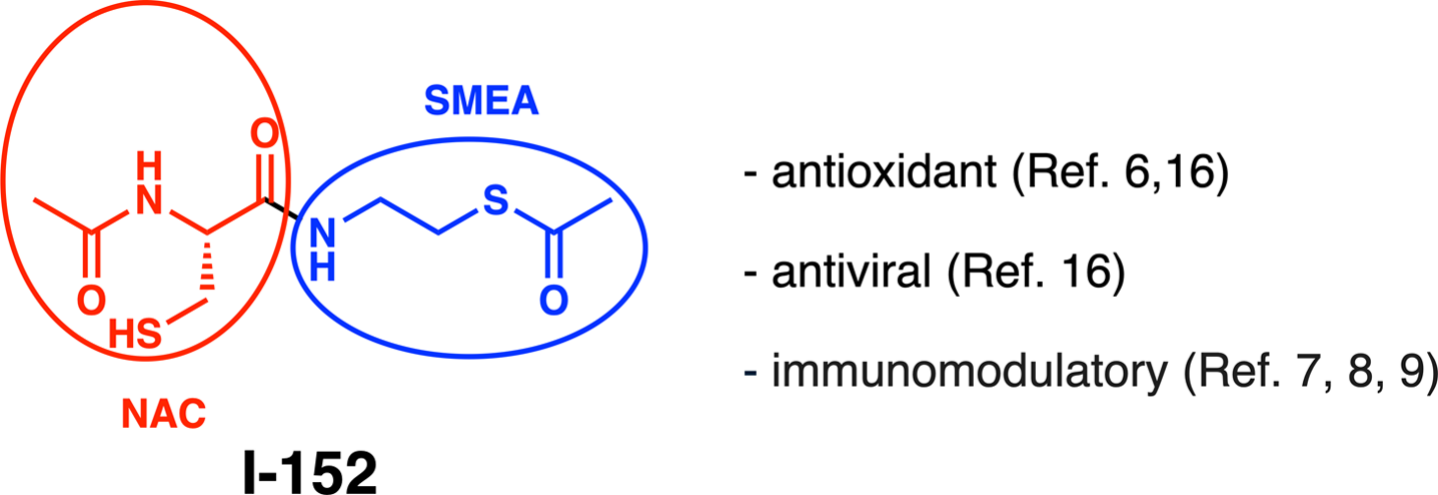
Structure and properties of I-152.

In addition, I-152 has two acetyl groups, forming amide and thioester moieties, which are common intermediates in biochemical processes and are expected to increase the lipophilicity, metabolic stability, bioavailability, and membrane permeability of the compound.^4^ I-152 maintains the acidic, ionizable, and reactive cysteine thiol. Total or partial deprotonation of the thiol enhances its nucleophilicity, thereby increasing its reactivity. This phenomenon underlies the key role of the cysteinyl residue in enzyme catalytic triads, where it acts as a nucleophilic center for electrophiles and serves as a target for oxidation by reactive oxygen species. Thus, both the high nucleophilicity of the free thiol/thiolate and the relatively high electrophilicity of the thioester are present together in the compound.

Once inside the cell, the compound undergoes enzymatic hydrolysis to release cysteamine- and cysteine-based intermediates, thereby boosting the intracellular thiol pool (Supporting Information).^5^

Biologically, I-152 exerts its antioxidant effect by strengthening endogenous defense mechanisms against oxidative stress through activation of the transcription factor Nrf2 and providing precursors for glutathione synthesis, i.e. Cys.^6^ I-152 has also been reported to have immunomodulatory activity, which is linked to its capacity to modulate immune responses both toward antigens and viral infections.^7–9^ Moreover, I-152 displays antiviral properties, which are thought to arise from the restoration of redox balance and the regulation of thiol-dependent cellular pathways that are critical for viral replication and host–virus interactions. Furthermore, it is a promising candidate for the treatment of conditions associated with glutathione depletion, oxidative stress, and immune dysregulation. Its mode of action has generally been assumed to be related to the thiol (or thiolate) site, which is the key moiety of the molecule. The reported thiolate-involved mechanisms of I-152 include free radical scavenging, rupture of disulfide bonds in cross-linked mucous proteins, metal complex formation, and acting as part of the glutathione redox system.

In this work, we delineate the ADME features and safety profile of I-152, defining how its distinctive thiol chemistry drives its biological fate and setting the stage for its advancement as a next-generation redox-modulating agent.

## 2 Experimental Procedures

### 2.1 Materials and Methods

I-152 (**1**) and NACMEAA (**2**) were synthesized as reported in the literature.^1^ ^1^H NMR spectra were recorded on a 400 MHz spectrometer, using CDCl_3_ and D_2_O, as solvents. Chemical shifts (δ scale) are reported in ppm relative to the central peak of the solvent. Coupling constants (*J* values) are given in Hz. LC-MS was performed using a Waters 2795 Separations Module and a Waters ZQ Mass spectrometer with a Gemini C6-Phenyl 110 A column (150 cm × 4.6 mm). The mobile phase consisted of 0.1% of formic acid in water (A) and acetonitrile (B). The LC gradient was as follows: 0–2 min, 2% B; 3–18 min, 2–90% B; 18–20 min, 90–95% B; the flow rate was 0.8 mL/min.

### 2.2 Synthesis of *S*-Acetyl I-152 (**3**)

To a solution of I-152 (**1**) (119 mg, 0.45 mmol) in acetonitrile (2.25 mL) were added acetyl chloride (32 μL, 0.45 mmol) and triethylamine (63 μL, 0.45 mmol). The reaction mixture was stirred at room temperature for 6 h. The solid obtained was removed by filtration, and the filtrate was diluted with DCM and water. The phases were separated, and the aqueous phase was further extracted with DCM. The combined organic phases were washed with brine and dried over anhydrous sodium sulfate, and the solvent was evaporated under reduced pressure. The residue obtained was crystallized with chloroform/petroleum ether to give **3** (66 mg, 48%) as a white solid. ^1^H NMR (400 MHz, CDCl_3_) δ 6.82 (s, 1H), 6.44 (d, *J* = 7.4 Hz, 1H), 4.57–4.52 (m, 1H), 3.46–3.41 (m, 2H), 3.33–3.23 (m, 2H), 3.08–2.98 (m, 2H), 2.38 (s, 3H), 2.37 (s, 3H), 2.02 (s, 3H). ^13^C NMR (100 MHz, CDCl_3_) δ 197.0, 196.2, 170.9, 170.1, 53.5, 39.7, 31.0, 30.7, 30.6, 28.6, 23.2.

### 2.3 Evaluation of Aqueous Solubility, Protein Binding, and Cell Permeability in Caco-2 Cells

The aqueous solubility, protein binding, and cell permeability of I-152 in Caco-2 cells were evaluated by Eurofins Panlabs (St. Charles, MO, USA).

Briefly, the aqueous solubility was evaluated in three types of media: simulated intestinal fluid, PBS (pH = 7.4), and simulated gastric fluid. I-152 was incubated at room temperature and a concentration of 2.0 × 10^–4^ M for 24 h. The aqueous solubility was determined by HPLC-UV/vis comparing the peak area of the principal peak in a calibration standard (200 μM) in organic solvent (methanol/water, 60/40, v/v) with the peak area of the corresponding peak in a buffer sample. The results shown are the mean of two independent experiments.

The protein binding of I-152 in human plasma was determined by incubating I-152 at a concentration of 1.0 × 10^–5^ M and 37 °C for 4 h. The sample was analyzed by HPLC-MS/MS, and the peak areas of the test compound in the buffer and in the test sample were used to calculate the percent binding and recovery.

Concerning the permeability in Caco-2 cells, I-152 was tested at 1.0 × 10^–5^ M after incubation with the cells for 60 min. The samples were analyzed by HPLC-MS/MS. The results shown are the mean of two independent experiments.

### 2.4 Evaluation of Half-life in Human Plasma and Intrinsic Clearance in Human Cryopreserved Hepatocytes

The half-life of I-152 in human plasma was determined by testing I-152 at a concentration of 1.0 × 10^–6^ M. The compound was incubated for 0, 30, 60, 90, and 120 min at 37 °C. The samples were analyzed by HPLC-MS/MS. The results shown are the mean of two independent experiments.

The intrinsic clearance in human cryopreserved hepatocytes was determined by testing I-152 at a concentration of 1.0 × 10^–6^ M. The compound was incubated for 0, 30, 60, 90, and 120 min at 37 °C. The samples were analyzed by HPLC-MS/MS. The results shown are the mean of three independent experiments.

### 2.5 Metabolic Stability in Human Liver Microsomes (HLMs)

Samples of I-152 at 10 μM in a 0.1 M phosphate buffer (PBS, pH 7.4) containing HLMs (0.5 mg/mL, pool of 10 donors; Merck cat. M0317) were incubated at 37 °C for 0, 5, 15, 30, and 60 min. The reactions were initiated by the addition of 1 mM NADPH. At each time point, an aliquot of reaction mixture was taken and quenched with ice-cold acetonitrile (containing 0.5 μM labetalol as an internal standard) at a 1:3 ratio. Proteins were precipitated by centrifugation at 12,000 rpm and 4 °C. The supernatants were analyzed by LC-MS/MS by Thermo Orbitrap. Blank sample was prepared by incubating the cell solution without any compound for 60 min. A reference incubation mixture (compounds in PBS) was prepared for each test compound and analyzed at *t* = 0 and 60 min in order to verify the compound’s stability in the mixture.

### 2.6 Metabolic Stability in Human Hepatocytes (HHs)

Cryopreserved hepatocytes, pooled from 10 donors (HMCS10, Lot. HUE125), were purchased from Thermo Fisher (Waltham, MA, USA). HHs were thawed, placed in a shaking water bath at 37 ± 1 °C, and purified using Cryopreserved Hepatocyte Recovery Medium (Thermo Fisher, CM7000). Hepatocytes were resuspended in Williams E medium (WEM), with supplements (Thermo Fisher, CM4000), and counted using trypan blue solution at a concentration of 0.5 × 10^6^ cells/mL. Samples with test compounds at 10 μM were incubated at 37 °C , and aliquots of 50 μL were collected at 0, 10, 30, 60, 120, and 240 min. The incubations were quenched at a 1:3 ratio with ice-cold acetonitrile (containing 0.5 μM labetalol as an internal standard). Samples were then centrifuged at 12,000 rpm and 4 °C for 10 min. The supernatants of centrifuged samples were analyzed by LC-MS/MS by Thermo Orbitrap, with the chromatographic method and MS settings reported below. The blank was prepared in a similar manner, but without the investigated compounds. A reference incubation mixture (compounds in WEM) was prepared for each test compound and analyzed at *t* = 0 and 240 min in order to verify the compound’s stability in the mixture.

### 2.7 UHPLC Coupled to HRMS for Metabolic Stability Studies

For each data set, chromatographic separation of metabolites was performed using a Thermo Ultimate 3000 UPLC system with a Phenomenex Luna Omega C18 column (1.6 μm, 2.1 × 150 mm) in positive polarity. The mobile phases consisted of 0.1% formic acid in water (A) and acetonitrile + 0.1% formic acid (B), respectively. The LC gradient was as follows: 0–4.5 min, 0–15% B; 4.5–12 min, 95% B; and 12–14 min, 95–0.5% B; the flow rate was 0.4 mL/min. Full MS scans were acquired with an Orbitrap Q-Exactive instrument over the range of *m*/*z* 100–800, with a resolution of 70,000, an automatic gain control (ACG) setting of 1 × 10^6^, and a maximum injection time of 100 ms. Peaks were fragmented in the high-energy collisional dissociation cell with a normalized collision energy of 30%, and the tandem mass spectrum was acquired in the orbitrap mass analyzer with a resolution of 17,500, ACG of 5 × 10^5^, and a maximum injection time of 50 ms. The full-scan MS/MS was a data-dependent acquisition (DDA) using a specific inclusion list generated through the software Mass-MetaSite 5.1.9 (MolDiscovery). The DDA method employed a minimum ACG target of 8 × 10^2^, an apex trigger of 1–2 s, isotopes excluded, and dynamic exclusion of 2 s.

### 2.8 hERG Assay

Human ether-à-go-go related gene (hERG) potassium channel was evaluated using an automated patch-clamp assay (IonChannelProfiler, Eurofins Discovery). Electrophysiological recordings were performed on CHO cells stably expressing the human hERG channel using the Qube automated patch-clamp platform. After establishing the whole-cell configuration, cells were held at −80 mV. hERG currents were elicited by depolarization to +40 mV for 500 ms followed by a ramp back to −80 mV over 100 ms to generate the characteristic tail current. This voltage protocol was applied every 8 s to monitor current amplitude. The peak tail current was measured before and after compound application. Percent inhibition of hERG current was calculated by comparing the current amplitude in the presence of the test compound to the control current amplitude. Compounds were tested in the presence of 0.1% Pluronic F-68 and at approximately room temperature.

### 2.9 Cell Culture for Toxicity Studies

Immortalized human keratinocytes (HaCat), human adenocarcinoma alveolar epithelial cells (A549), and immortalized human embryonic kidney cells (HEK293T) were used. Cells were grown in high-glucose Dulbecco’s modified Eagle’s medium (Euroclone, Italy), supplemented with 10% fetal bovine serum, 2 mM glutamine, 100 μg/mL streptomycin, and 100 μg/mL penicillin. The cell culture medium was changed every 2 days, and passage was done at 80% confluency after detachment of cells by (i) trypsin (0.25%)-EDTA for HaCat cells, (ii) trypsin (0.05%)-EDTA for A549 cells, and (iii) 1% PBS for HEK293T cells. All cell lines were maintained in an incubator at 37 °C and 5% CO_2_.

### 2.10 Lactate Dehydrogenase (LDH)-Based Cytotoxicity Assay

The release of LDH was used as a marker for cell integrity to assess the cytotoxic potential of cells exposed to I-152 treatment. HaCat, HEK 293T, and A549 cells (50,000 cells/well) were treated with increasing concentrations of I-152 (0.06, 0.25, and 1 mM) for 24 h; each condition was assayed in duplicate, and three independent experiments were performed. The LDH activity was measured in a spectrophotometer (340 nm) for 10 min, monitoring the NADH consumption and following the protocol described by Beutler.^10^ Cell culture medium alone was used as a blank, and the OD values were subtracted from the readings for each condition.

### 2.11 Microscopic Assessment and Flow Cytometric Analysis

Cells were seeded at 400,000 cells/well in a 6-well plate before being treated with increasing concentrations of I-152 (0.06, 0.25, and 1 mM) for 24 h. Prior to flow cytometric analyses, an inverted microscope (Olympus IX 51, Olympus Corporation, Japan) equipped with a ToupTek camera and combined with ToupTek software (ToupTek Photonics, China) was used for cell plate imaging under a bright-field light (10× magnification).

Propidium iodide (PI) is a membrane-impermeant DNA-binding dye that cannot penetrate viable cells. Hence, PI allows the detection of necrotic or apoptotic cells with compromised plasma membranes. Cells were detached as described above and resuspended in PBS, to which 1.5 μL of PI (10 g/mL) (Sigma-Aldrich, Germany) was added 10 min before flow cytometric analysis. The samples were analyzed using the phycoerythrin fluorescence channel in a CytoFlex V3B3R3 flow cytometer (Beckman Coulter, Netherlands). Cell death analysis (% of PI^+^ cells) was performed with CytExpert software (version 2.6, Beckman Coulter, Netherlands); approximately 10,000 cell events were acquired for each sample. The results are from three independent experiments.

### 2.12 Statistical Analysis

Statistical comparisons were performed using one-way ANOVA analysis with Dunnett’s posthoc test with GraphPad Prism, version 10.05.00 software (*p* < 0.05 indicated statistical significance).

## 3 Results and Discussion

### 3.1 Organic Chemistry Features of I-152 (**1**)

I-152 (**1**) displays appreciable solubility in both aqueous and organic media, facilitating its manipulation under a variety of experimental conditions and enabling its potential application in diverse biological and chemical assays. In fact, although thioesters serve as good acyl donors, e.g., native chemical ligation,^11,12^ they are characterized by a relatively slow rate of hydrolysis, a kinetic feature/phenomenon that distinguishes them from their comparable activated carboxylic acid. The reduced reactivity in aqueous media enables their use as stable yet reactive intermediates and substrates in synthetic and biochemical transformations under physiological conditions. This unique property allows sulfur-containing compounds to be used as substrates in aqueous environments. One of the most well-known thioesters is acetyl coenzyme A, which plays a crucial role in various biochemical processes such as the citric acid cycle, and other thioesters are involved in polyketide biosynthesis.

While I-152 (**1**) was found to be stable for more than 48 h in CDCl_3_ (Supporting Information), we observed a rapid transposition of I-152 (**1**) in D_2_O and in CD_3_OD, giving rise to the corresponding dithiol **2** and fully acetylated derivative **3** (Scheme 1) (Supporting Information). The structures of the transposition products were confirmed by HPLC/MS and comparison with analysis of authentic synthesized samples (Supporting Information). An identical result was also observed when I-152 (**1**) was incubated with total cell extracts.^13^ The fact that this transposition was markedly influenced by the solvent and the concentration strongly points to the participation of an intermolecular process, rather than a purely intramolecular event.

**Scheme 1:**
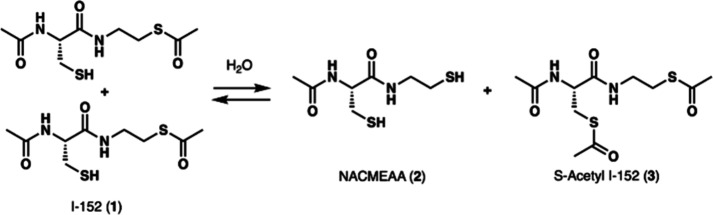
Transposition of I-152 (**1**)

HPLC-MS analysis also indicated that NACMEAA (**2**) and *S*-acetyl I-152 (**3**) remained stable for several hours under the same neutral aqueous conditions (100 mM phosphate buffer, pH 7.4, 37 °C) (Supporting Information). These findings suggest that *S*-acetylated species have a high electrophilicity, while maintaining sufficient aqueous stability. This balance facilitates intermolecular acyl transfer in water.

This unanticipated phenomenon may nonetheless offer beneficial implications despite making some assays more difficult to perform (e.g., the plasma stability assay). In fact, the equilibrium governing I-152 can be viewed as a dynamic strategy to enhance its delivery, whereby transient modification of physicochemical properties facilitates membrane permeation.^14^ This temporary adjustment is subsequently reversed through efficient enzymatic cleavage in vivo, thereby releasing the parent molecules at the intended site of action.^15^ Notably, the dithiol derivative NACMEAA may represent a key contributor to the mode of action of I-152, as its disulfide-reducing capability, together with its favorable (bio)­chemical profile, can critically influence the overall pharmacological outcome.^1,16^ In detail, the thioester of one I-152 molecule reacts with the free thiol of another I-152 molecule, leading to the transfer of the acyl group from one sulfur atom to another, i.e. transthiolation (also known as transthioesterification). Transthiolation relies on the formation and breaking of thioester bonds, which are high-energy bonds that facilitate the transfer of large molecules. In fact, this type of reaction is used in the biosynthesis of acetyl coenzyme A, fatty acids, and polyketides, as well as for post-translational modification by the ubiquitin system.^17–19^ The mechanism by which thioester bonds undergo isoenergetic transfer in both directions—resulting in an equilibrium where all three species coexist—is not fully understood, particularly regarding the influence of water or other protic solvents like methanol. Furthermore, the reactivity of thioesters stands out due to their unique behavior. Interestingly, thioesters have been reported to be significantly more reactive than oxoesters when interacting with nonoxygen nucleophiles, such as amines and thiolates.^20,21^ These general observations were in this fast intermolecular transposition in water, clearly demonstrating the strong attitude of the thioester to react with another thiol. To better understand these reactivity patterns and to make comparisons between reactions with different biological nucleophiles, we also explored the reactivity with alkyl primary amine (mimic lysine) and alcohol/phenol (mimic serine/threonine and tyrosine). Reaction of I-152 with neutral amine such as benzylamine rapidly and quantitatively afforded the *N*-acetylated product, whereas no reactions occurred with ethanol or the biologically interesting compound tyrosol. The rates of transposition of I-152 in the presence of protonated or *N*-acetyl benzyl amine were comparable to their aqueous stabilities, suggesting the low reactivity of I-152 toward Lys residues under neutral aqueous conditions. Accordingly, the reaction of I-152 with the less-nucleophilic *p*-aminophenol was sluggish, and the LC-MS analysis revealed partial monoacetylation after 72 h. These latter studies provide information on aminolysis reactions of thioesters, their reactivity with oxygen-based nucleophiles, as well as their hydrolytic stability and selectivity.

Recently, several groups have reported that Cys acylation with small, strongly electron-withdrawing acyl groups, such as formyl, acetyl and α-fluoroacetyl groups, would effectively induce hydrolysis of the adjacent amide bond (cleavage of the amide bond in the i-1 position to the acylated Cys residue) under neutral aqueous conditions via an intramolecular thioester-to-imide acyl transfer step.^22–24^ In our case, neither S-to-N acyl transfer nor cleavage of the main-chain amide bonds in I-152 was observed under neutral aqueous conditions. However, as will be detailed below, I-152 demonstrated a measurable instability toward proteases and peptidases in vivo, a feature that may critically influence its pharmacokinetic behavior (Scheme 2).

**Scheme 2:**
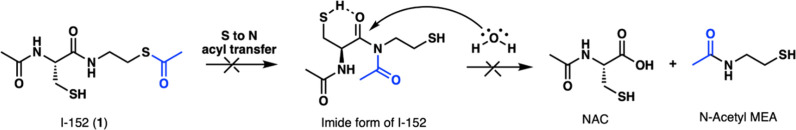
S-to-N Acetyl Transfer of I-152 (**1**) and Amide Bond Hydrolysis of the Imide Intermediate

### 3.2 Aqueous Solubility, Permeability, and Protein Binding

The aqueous solubility, protein binding, and Caco-2 cell permeability of I-152 (**1**) were evaluated by Eurofins Panlabs (St. Charles, MO, USA) (see the Section 2), and the results are shown in Table 1.

**Table 1:** Aqueous Solubility, and Cell Permeability of I-152

**I-**152 (1)	
aqueous solubility in simulated intestinal fluid	>200 μM
aqueous solubility in PBS, pH 7.4^a^	69.33 μM
aqueous solubility in simulated gastric fluid	>200 μM
A-B permeability (Caco-2, pH 6.5/7.4)	1.4 × 10^–6^ cm/s (56%)^b^
B-A permeability (Caco-2, pH 6.5/7.4)	0.9 × 10^–6^ cm/s (68%)^b^

a The ionic strength is maintained with 137 mM NaCl.

b % of recovery is provided in brackets.

I-152 displayed a good aqueous solubility in PBS at pH = 7.4. When tested in biorelevant media such as simulated gastric or intestinal fluid, a significant increase in solubility was observed, as expected.^25^

Plasma protein binding could not be assessed due to rapid I-152 instability likely leading to concentrations below the limit of detection. Indeed, the compound was detected in control samples at time = 0. It was hypothesized that during the incubation, the compound underwent protein-promoted hydrolysis, since analysis by triple-quadrupole mass spectrometry did not detect any other products. This hypothesis was subsequently validated by detection of different thiol species, e.g. cys, NAC and MEA, after 10 min of I-152 incubation in human plasma as discussed below and reported in Supporting Information.

The apparent permeability (*P*_app_) of I-152 was assessed in Caco-2 cells by measuring transport in both directions: apical-to-basolateral (A→B) and basolateral-to-apical (B→A). A→B permeability reflects passive diffusion, paracellular permeation, and active transport, while B→A permeability provides insight into transporter-mediated efflux. When the A→B and B→A values are similar, passive diffusion or paracellular permeation predominates (e.g., nonsaturated conditions). In contrast, a significant deviation from 1 indicates some contribution of active transport. For example, when the efflux ratio (ER) is greater than or equal to 2 (ER = *P*_app(B→A)_/*P*_app(A→B)_), active efflux is likely to occur.^26^ For I-152, the observed ER was 0.64, suggesting that active uptake is likely and active efflux is minimal. However, the low recovery values indicate that I-152 may undergo degradation or biotransformation within Caco-2 cells. I-152 is metabolized intracellularly as reported below, making the interpretation of these data uncertain.

### 3.3 In Vitro Metabolism

First, the stability of I-152 in human plasma was explored at several time points (0, 30, 60, 90, and 120 min). The half-life of I-152 was determined to be 8.9 min (Figure 2A).

**Figure 2:**
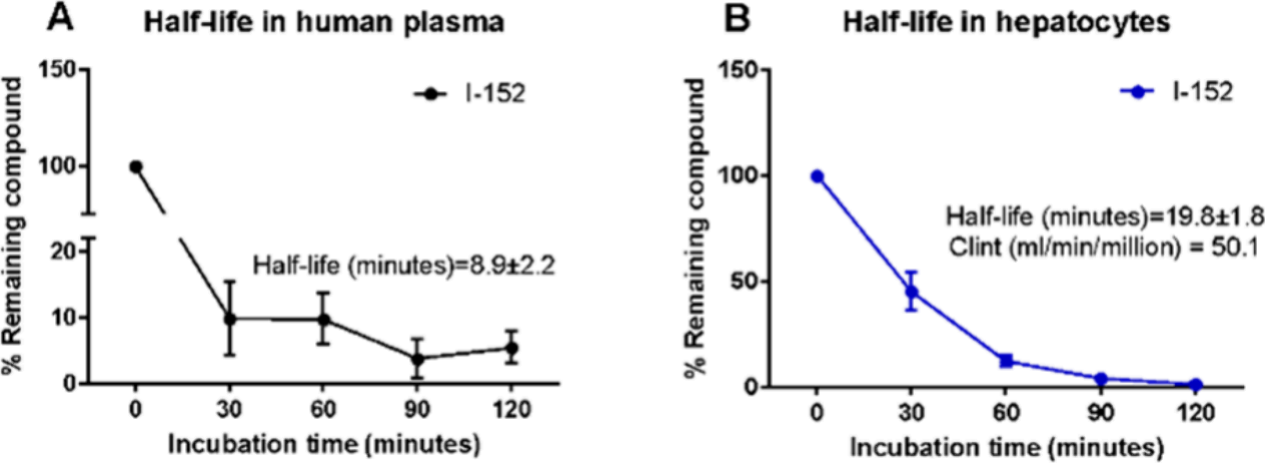
Stability of I-152 in human plasma (A) and in human cryopreserved hepatocytes (B). The assays were performed at Eurofins Panlabs USA (see the Section 2).

At the same time points, hydrolysis products and increased Cys levels were measured through the 5,5′-dithio-bis­(2-nitrobenzoic acid)-HPLC method developed for the individual identification and quantification of biologically important thiols (biothiols) in complex matrices such as biological fluids, cells, and tissues (Supporting Information).

In cryopreserved HHs, the half-life of I-152 was 19.8 min, with an intrinsic clearance of 50.1 μL/min/million cells) (Figure 2B). To identify thiol derivatives of the intracellular metabolism of I-152, further studies were performed in HLMs and in cryopreserved HHs.

Samples were analyzed by LC-MS/MS and raw data were processed using MassMetaSite 4.5.0–2 in “the WebMetabase application, encapsulated in the web platform Oniro version 1.5.3 (Mass-Analytica TM).^27,28^

In HLMs, I-152 was incubated for 60 min. It was observed that the amount of I-152 decreased over time until complete disappearance, and a half-life of approximately 10 min was calculated (Figure 3A). Along with the parent compound, two thiol derivatives resulting from its disproportionation were identified by comparing their *m*/*z* and RT with standard compounds (Supporting Information), exhibiting similar kinetics: NACMEAA and its triacetylated derivative (Scheme 1). Additionally, the formation of a compound with *m*/*z* 221.0410 was observed, which is consistent with the cyclic oxidized forms of NACMEAA previously reported by Bartoccini et al.^1^ Under our experimental conditions, however, neither NAC nor MEA—which can be formed through hydrolysis of NACMEAA—were detected due to their low molecular weight. However, in parallel experiments conducted in different cellular models by using the above cited HPLC method, NAC and MEA were always found and quantified.^6,16^ To investigate the possible involvement of phase II reactions, I-152 was incubated for 4 h with HHs in WEM (Figure 3B). In this medium, I-152 could not be detected, while the triacetylated derivative, identified by *m*/*z* and RT comparing it with the standard mixture (Supporting Information), was visible only at time 0. The compound with *m*/*z* 221 was present and remained constant over time, suggesting that NACMEAA may exist in its cyclized form. Interestingly, a metabolite with *m*/*z* 251.0877 was also detected; its fragmentation pattern was analyzed and found to be consistent with double methylation of the thiol groups of NACMEAA (Figure 4).

**Figure 3:**
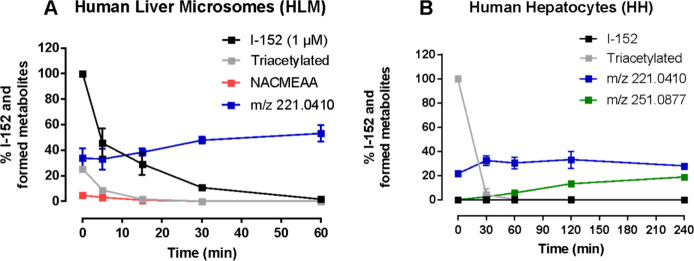
Metabolic stability and metabolite analysis of I-152 in HLMs (A) and HHs (B). I-152 was incubated with HLMs for 60 min and with HHs for 4 h, respectively. At predetermined time points, the reaction was stopped, and the samples were analyzed by LC-MS/MS. The results shown are the mean ± standard deviation of at least two independent experiments.

**Figure 4:**
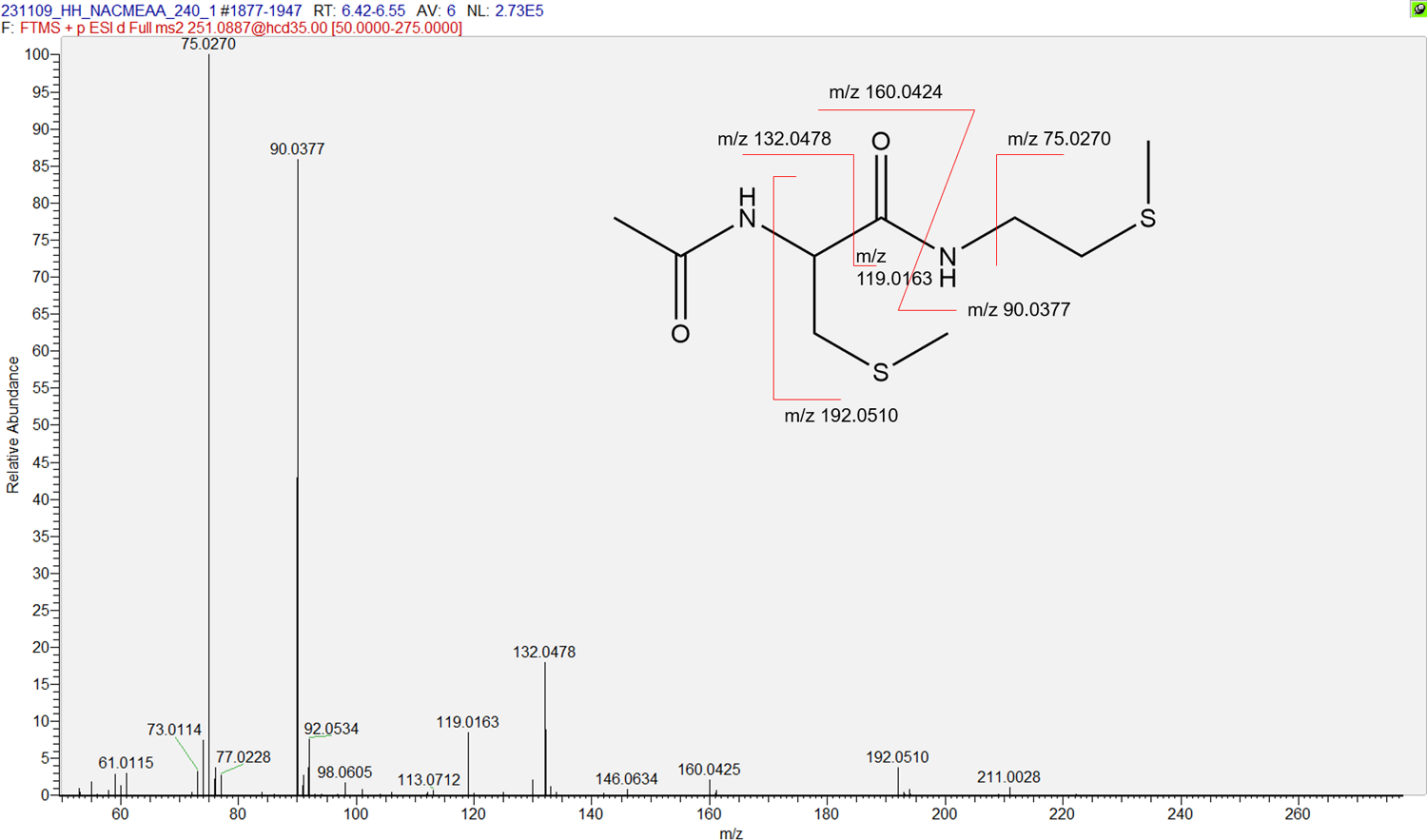
MS/MS spectrum of the compound with *m*/*z* 251 and assignment of the major fragments.

To understand why I-152 could not be detected in the hepatocyte experiments, the compound was incubated in WEM alone, without hepatocytes. Even under these conditions, I-152 was not detectable, due to its instability while both its triacetylated (S-Acetyl I-152 (**3**)) and the cyclic oxidized forms of NACMEAA *(* *m*/*z* 221) were identified by RT and remained stable over time (data not shown). Moreover, it is conceivable that I-152, because of its instability, was partly hydrolyzed in the medium due to the presence of serum; however, intracellular I-152 detection was accomplished up to 24 h in other cellular models, such as RAW 264.7 and THP-1 cells incubated with the compound as previously reported^6,9^ by using the HPLC method cited above. To further confirm that the *m*/*z* 251 metabolite was related to NACMEAA, the latter was incubated under the same experimental conditions with HHs (Figure 5). Once again, NACMEAA was detected only in its *m*/*z* 221 form and showed disappearance kinetics consistent with its metabolism, with a half-life of approximately 130 min. The main metabolite was the compound with *m*/*z* 251, observed at the same retention time as in the I-152 experiments, suggesting that this metabolite with a mass difference of +28, can derive from NACMEAA. Two additional metabolites with a mass difference of +14 (*m*/*z* 237.0727) were also detected, although MS/MS spectra were not acquired in this untargeted approach. Notably, these +14 metabolites exhibit increasing kinetics over time, which is typical of metabolite formation.

**Figure 5:**
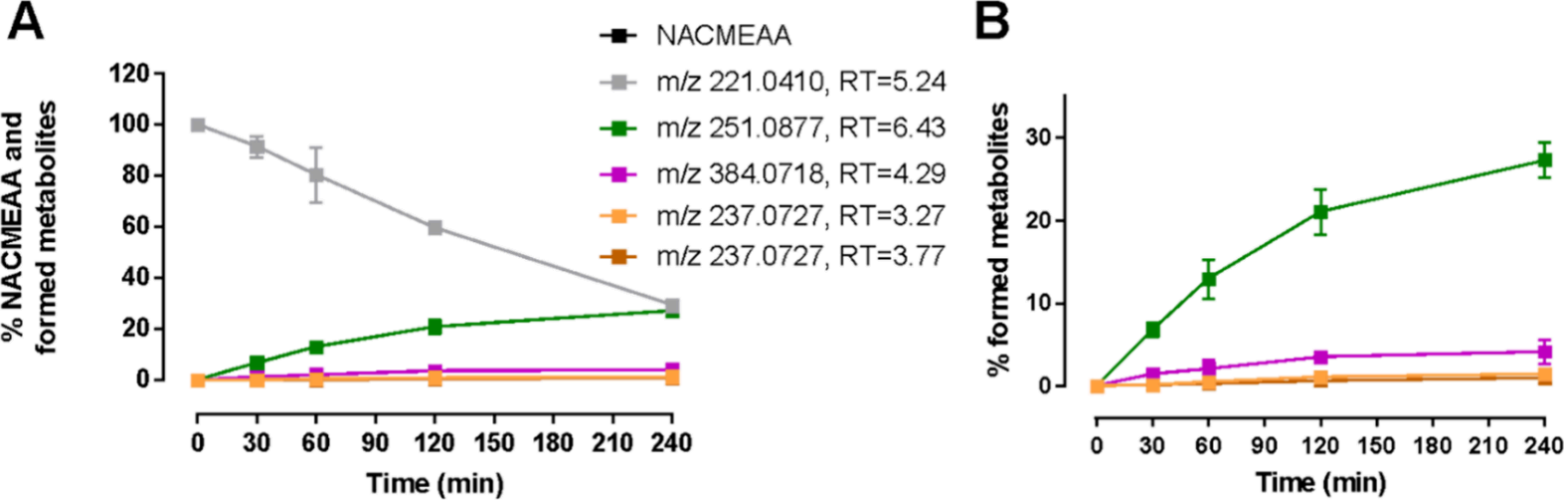
Metabolic stability of NACMEAA in HHs. % of NACMEEA and formed metabolites (A) and formed metabolites (B). NACMEAA was incubated with HHs for 4 h. At predetermined time points, the reaction was stopped, and the samples were analyzed by LC-MS/MS. The results shown are the mean ± standard deviation of at least two independent experiments.

Although at the speculative level, we reasoned that S-methylation reactions in nature are carried out by *S*-adenosylmethionine (SAM)-dependent methyltransferases, such as thiol methyltransferase or thiopurine methyltransferase. The coenzyme SAM is synthesized by the enzyme methionine adenosyltransferase from ATP and methionine. In HHs, where the hepatocyte remains intact, it is possible to observe the formation of metabolites resulting from such methylation reactions. In microsomes, however, although the responsible enzymes are present—as they are localized in the endoplasmic reticulum—activation of these reactions would require the addition of the SAM coenzyme.^29^ Certainly, further studies to prove this assumption will be needed.

Finally, an additional NACMEAA metabolite with *m*/*z* 384.0718 (M+161) was also detected. This metabolite might result from conjugation of NACMEAA with NAC, but no MS/MS spectra are available to confirm this hypothesis. The synthesis of more stable, nonhydrolyzable I-152 analogues will allow us to establish whether the observed biological effects are attributable to I-152 itself or to its released metabolites.

### 3.4 Toxicity Studies

I-152 was evaluated for inhibition of the hERG potassium channel using the CiPA automated patch-clamp assay. The compound showed weak inhibition of hERG current across the tested concentration range (0.1–100 μM), with maximal inhibition below 20%, and therefore no calculable IC_50_ value was obtained (Supporting Information). Drug discovery deeply relies on cell viability studies to investigate the potential toxic effects of drug candidates. The present investigation included classical toxicological criteria, i.e., cell viability analysis and morphological parameters. In vitro toxicity tests to assess the safety of I-152 were performed on three mammalian cultured cell lines. HaCaT cells are immortalized human keratinocytes and have been extensively used to study epidermal homeostasis and pathophysiology.^30^ A549 cells are adenocarcinomic human alveolar basal epithelial cells widely used for both basic research and drug discovery.^31,32^ Finally, HEK293T are epithelial-like cells that were isolated from the kidney of a patient. These cell lines have the ability to grow continuously in cell culture, and they provide a useful platform to investigate the cellular mechanisms of pathogens such as viruses.^33^ Cell viability was determined by PI staining (Figure 6A) and by LDH dosage experiments (Figure 6B). PI is a DNA-binding dye that cannot penetrate the membranes of living cells, making it useful for identifying dead or damaged cells in viability assays. LDH is a cytoplasmic enzyme that catalyzes the concomitant interconversions of pyruvate to L-lactate and NADH to NAD^+^ during fermentation, and the reverse reactions during the Cori cycle.^34,35^ In response to cellular damage, LDH is released from the cytoplasm into the extracellular environment. Its stability in cell culture medium makes it a well-matched marker for the presence of damage and toxicity in tissues and cells.^36^ The doses to test I-152 toxicity were selected based on those previously used for NAC and MEA in in vitro studies.^37,38^ After a 24 h exposure to increasing concentrations of I-152, both cell viability assays yielded comparable results: 1 mM I-152 resulted in approximately 15% of membrane-damaged cells across all three cell lines tested (Figure 6). No changes in the cell morphology of cells treated with 0.06 mM or 0.25 mM I-152 were observed (Figure 6C; only those treated with 0.06 mM I-152 are shown), while those treated with 1 mM I-152 were slightly rounded, likely as a result of cytoskeletal derangement. Another mechanism by which I-152 may cause cellular dysfunction could be correlated with the excessive amount of reducing equivalents in the presence of intact oxidoreductive systems inducing reductive stress. As already observed with other antioxidant thiols, I-152 may activate signaling pathways that target respiratory complexes of the mitochondrial electron transport chain which are implicated in ROS production.^39^ Under reductive stress, abnormal formation of disulfide bonds activates the unfolded protein response (UPR) and induces endoplasmic reticulum stress altering protein structure and functions.^40^ These results, together with those previously obtained in repeated-dose studies using 300 mg/kg I-152,^7,8^ provide an initial understanding regarding the toxicity profile of I-152. Comparative studies with NAC and MEA were conducted by using PI on only the HEK293T cell system given the similar results obtained in the three cell lines. We found that toxicity profile of I-152 was similar to the single compounds (Supporting Information). MEA is rapidly oxidized in air or aqueous solution to its disulfide form cystamine and its toxicity has been previously correlated to H_2_O_2_ generation and glutathione peroxidase inhibition.^41,42^ NAC at high concentrations might have the capability to reduce disulfide bridges in proteins resulting in structure and/or function alteration.^43^ In conclusion, combining NAC and MEA into I-152 allowed obtaining a more potent antioxidant molecule without altering toxicity of single compounds.

**Figure 6:**
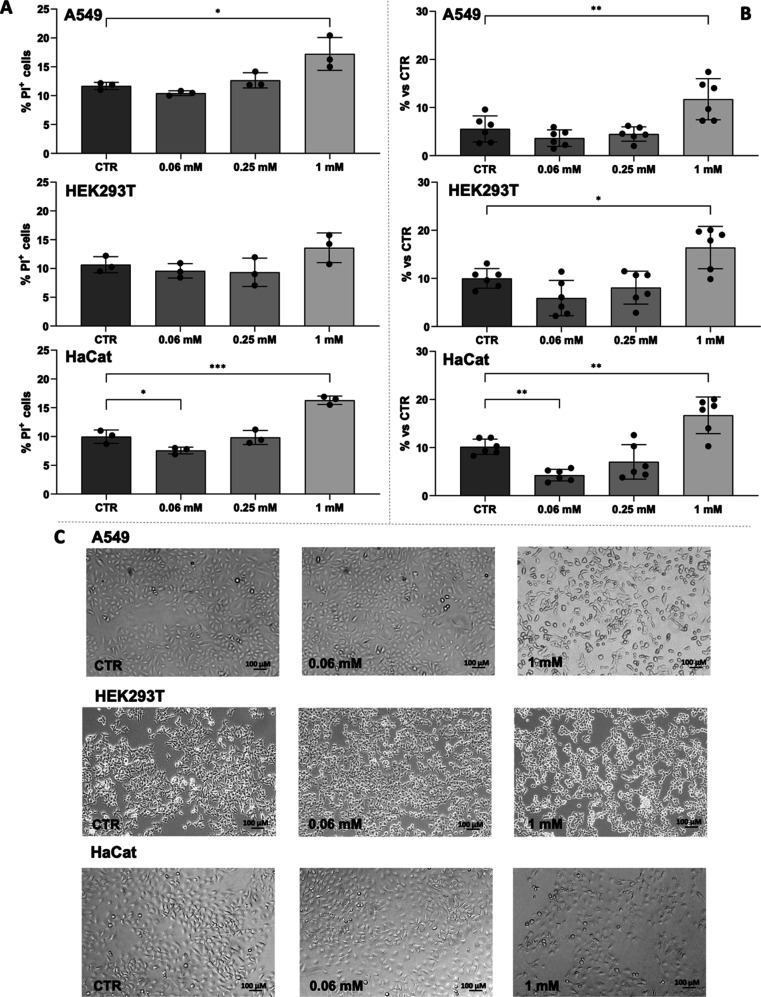
I-152 cytotoxicity. Three different cell lines were treated with increasing I-152 concentrations, and the cell viability was assayed at 24 h by spectrophotometrically (340 nm) measuring the LDH activity according to the protocol described in Beutler.^10^ (A) and by propidium iodide (PI) uptake using flow cytometry (B). Data are reported as % PI-positive cells or as the fold change relative to control (CTR). The scatter dot blots, which include the mean and standard deviation error bars, represent independent experiments, and each point is the mean of two technical replicates. Asterisks indicate statistically significant results. *p < 0.05, **p < 0.01, and ***p < 0.001.

In conclusion, this work provides an initial ADME and safety profile of I-152, a NAC–cysteamine conjugate with redox-modulating properties. The compound shows good solubility and acceptable Caco-2 permeability, while its limited plasma and microsomal stability is consistent with the formation of multiple metabolites, including NACMEAA and related metabolites. These species may contribute to the observed overall biological effects and suggest a prodrug-like behavior. Toxicity assays in three human cell lines showed minimal cytotoxicity up to 0.25 mM and modest effects at 1 mM, indicating low acute cytotoxicity under the tested in vitro conditions. Overall, I-152 appears a chemically versatile thiol donor (highly labile precursor of pro-species), while additional studies, including more stable analogues, are needed to clarify the roles of the parent compound and its metabolites.

## Supplementary Material



## Notes

Copies of ^1^H NMR spectra of compounds I-152 (**1**), NACMEAA (**2**), and *S*-acetyl I-152 (**3**); Copies of ^13^C NMR spectra of *S*-acetyl I-152 (**3**); copies of the HPLC chromatograms of I-152 (**1**) and NACMEAA (**2**); copy of the HPLC-MS chromatogram of the reaction between I-152 (**1**) and benzylamine; NAC, MEA, and Cys detection in human plasma incubated with I-152 (1 mM); EIC chromatograms of thiols standards and I-152 incubated in HLM and HH; hERG assay and comparative toxicity studies between I-152 and NAC or MEA­(PDF)

## ABBREVIATIONS

A549: human adenocarcinoma alveolar epithelial cells
ACG: automatic gain control
DDA: data-dependent acquisition
ER: efflux ratio
HaCat: immortalized human keratinocytes
HEK293T: immortalized human embryonic kidney cells
HHs: human hepatocytes
HLMs: human liver microsomes
LDH: lactate dehydrogenase
NAC: *N*-acetyl-l-cysteine
PI: propidium iodide
SMEA: *S*-acetyl-β-mercaptoethylamine
SAM: *S*-adenosylmethionine
WEM: Williams E medium
