# Supplementary material for: Early ADME and Preliminary Toxicity Studies of I‑152, an N‑Acetyl‑l‑Cysteine/S‑Acetylcysteamine Conjugate
Source: Chem Res Toxicol. 2026 Apr 27;39(5):929–38. doi: 10.1021/acs.chemrestox.6c00018 (PMC13188162; doi:10.1021/acs.chemrestox.6c00018)
Supplement: Supplementary file 1 [file tx6c00018_si_001.pdf]

SUPPORTING INFORMATION

Early ADME and Preliminary Toxicity Studies of I-152, an *N*-Acetyl-L-Cysteine/*S*-Acetylcysteamine Conjugate

Francesca Bartoccini,[a], *Aurora Valeri,*[c] *Matteo Gregori,*[a] *Sofia Masini,*[a] *Michela Bruschi,*[a] *Laura Goracci,*[b] *Alessandra Fraternale,*[a,*] *Giovanni Piersanti*[a,*]

[a]Department of Biomolecular Sciences, University of Urbino Carlo Bo, Via Ca' Le Suore 2, Urbino 61029, Italy.

[b]DAISY Lab (Drug Discovery-Artificial Intelligence-Organic Synthesis), Department of Chemistry, Biology and Biotechnology, University of Perugia, Via Elce di Sotto 8, Perugia 06123, Italy.

[c]Molecular Horizon srl, Via Montelino 30, Bettona 06084, Italy.

*Correspondence to:
Email: giovanni.piersanti@uniurb.it and alessandra.fraternale@uniurb.it

Table of Contents

Figure S1. ^{1}H NMR spectrum (400 MHz) of I-152 (**1**) in $CDCl_3$ S3

Figure S2. ^{1}H NMR spectrum (400 MHz) of I-152 (**1**) in D_2O S3

Figure S3. ^{1}H NMR spectrum (400 MHz) of NACMEAA (**2**) in $CDCl_3$ S4

Figure S4. ^{1}H NMR spectrum (400 MHz) of NACMEAA (**2**) in D_2O S4

Figure S5. ^{1}H NMR spectrum (400 MHz) of S-Acetyl I-152 (**3**) in $CDCl_3$ S5

Figure S6. ^{1}H NMR spectrum (400 MHz) of S-Acetyl I-152 (**3**) in D_2O S5

Figure S7. ^{13}C NMR spectrum (100 MHz) of S-Acetyl I-152 (**3**) in $CDCl_3$ S6

Figure S8. HPLC chromatogram of I-152 (**1**) a t = 0 s S7

Figure S9. HPLC chromatogram of I-152 (**1**) a t = 4 h S8

Figure S10. HPLC chromatogram of I-152 (**1**) a t = 20 h S9

Figure S11. Comparison of HPLC chromatogram of I-152 (**1**) at t = 0 s, t = 4 h and t = 20 h S10

Figure S12. HPLC chromatogram of NACMEAA (**2**) S11

Figure S13. MS chromatogram of NACMEAA (**2**) S12

Scheme S1. Reaction of I-152 (**1**) with benzylamine,tyrosol and *p*-amino phenol S13

Figure S14. HPLC-MS chromatogram of the reaction between I-152 (**1**) and benzylamine S13

Scheme S2. Hydrolysis of I-152 (**3**) S14

Figure S15. NAC, MEA and cys detection in human plasma incubated with I-152 S14

Figure S16. Representative overlaid extracted ion current (EIC) chromatograms of a mixed standard solution (A) and a sample of I-152 incubated in HLM S15

Figure S17. Representative overlaid extracted ion current (EIC) chromatograms of a mixed standard solution (A) and a sample of I-152 incubated in HH. S16

Table S1. hERG assay S17

Figure S18. Comparative toxicity studies between I-152 and NAC or MEA S18

References. S18

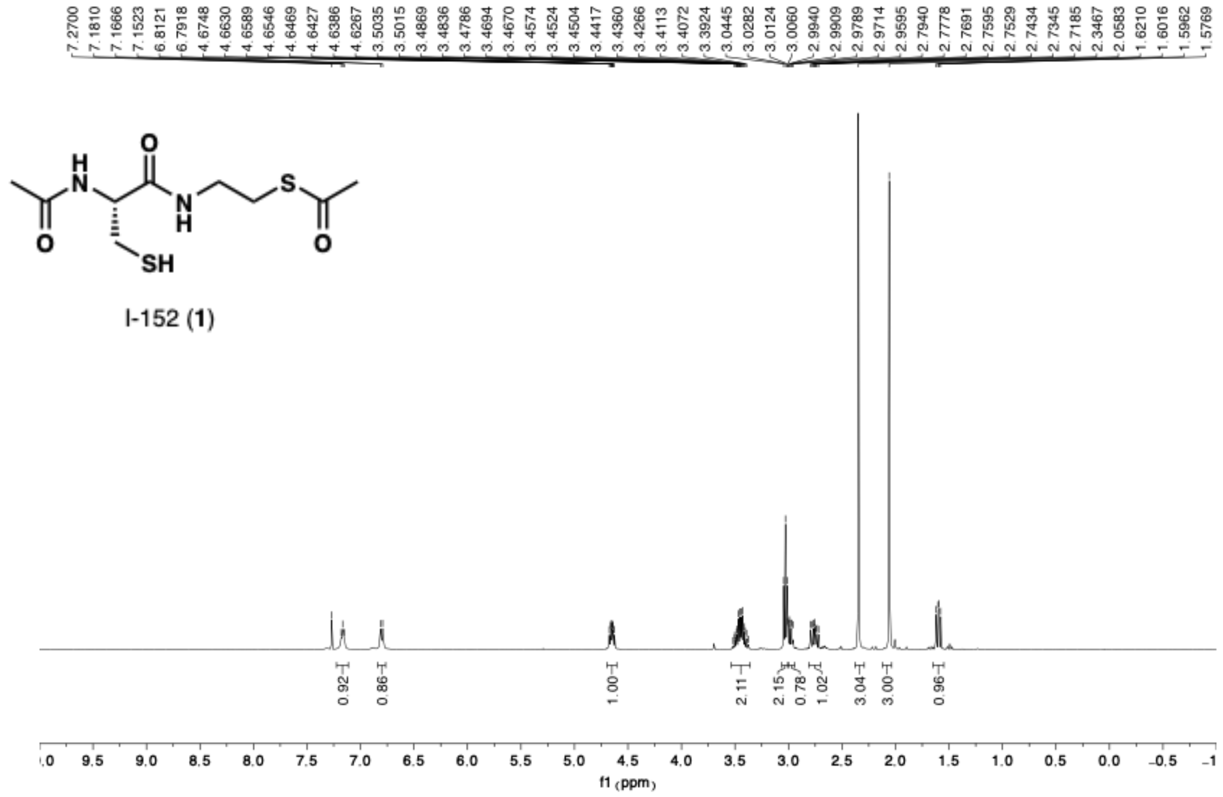

Figure S1. ^{1}H NMR spectrum (400 MHz) of compound of I-152 (**1**) in $CDCl_3$. The compound is stable for a long time (more than 48 hours).

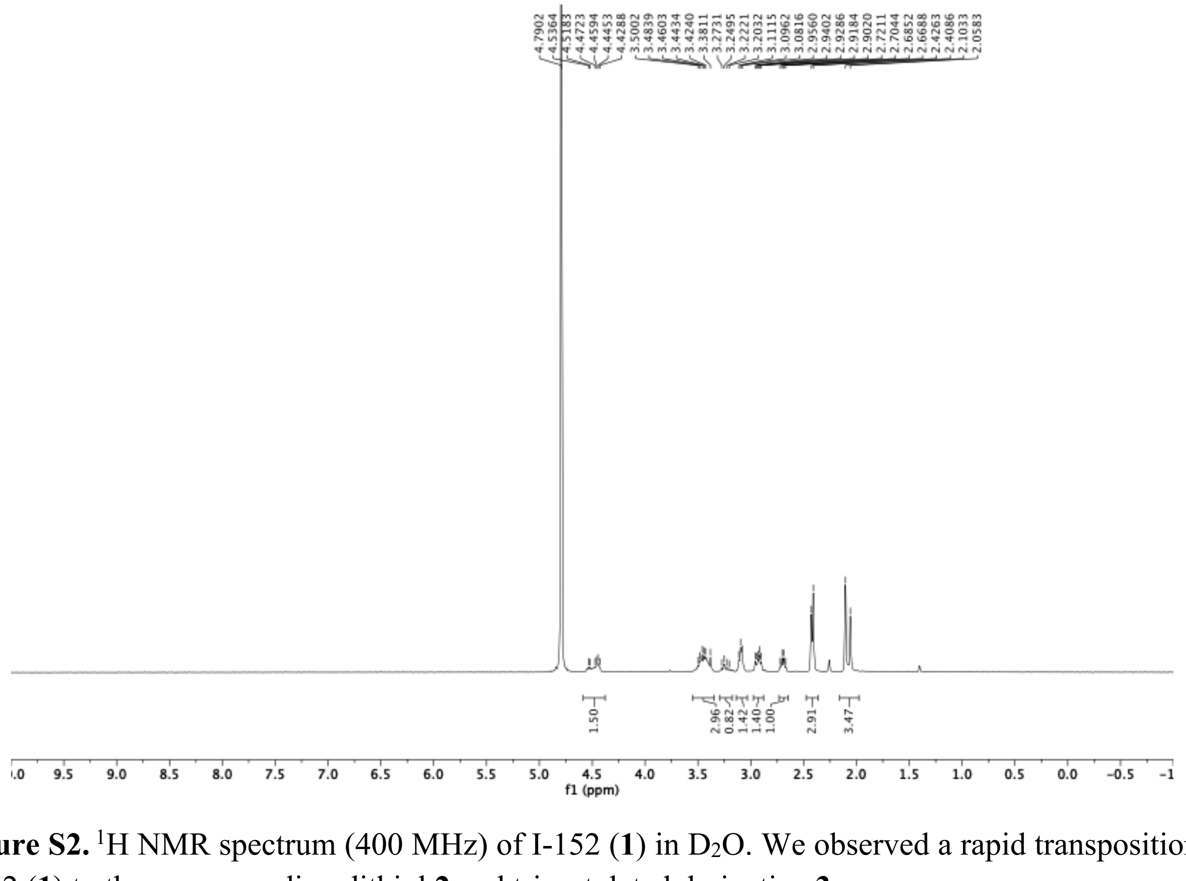

Figure S2. ^{1}H NMR spectrum (400 MHz) of I-152 (**1**) in D_2O. We observed a rapid transposition of I-152 (**1**) to the corresponding dithiol **2** and triacetylated derivative **3**.

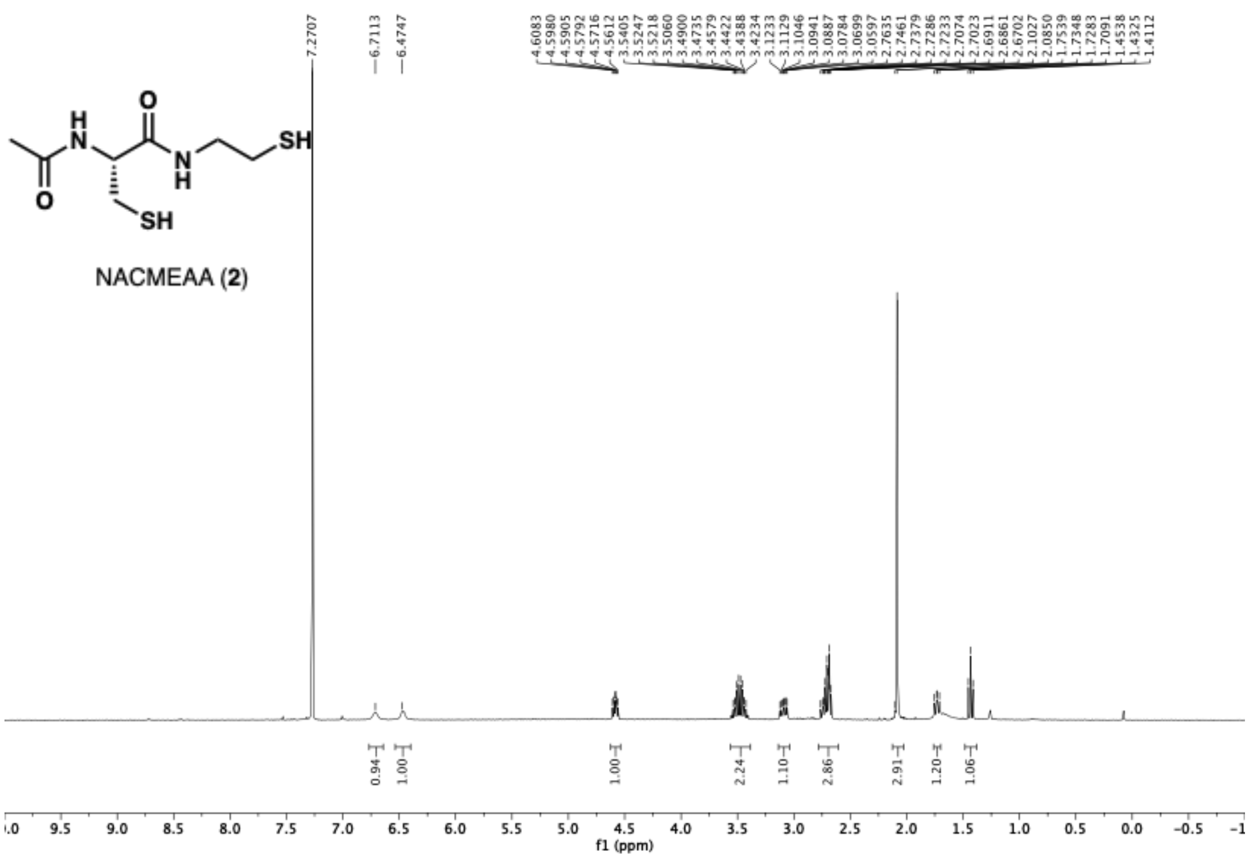

Figure S3. ^1H NMR spectrum (400 MHz) of NACMEAA (**2**) in $CDCl_3$.

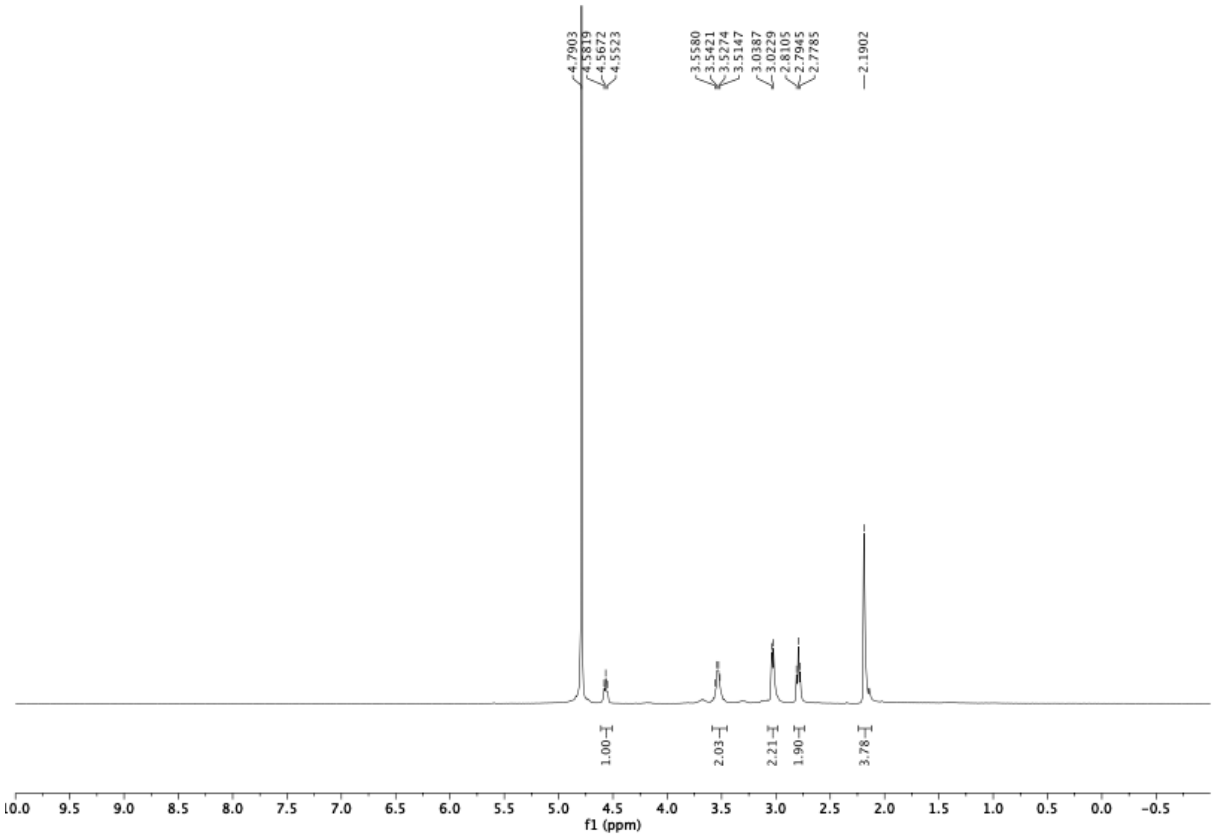

Figure S4. ^1H NMR spectrum (400 MHz) of NACMEAA (**2**) in D_2O.

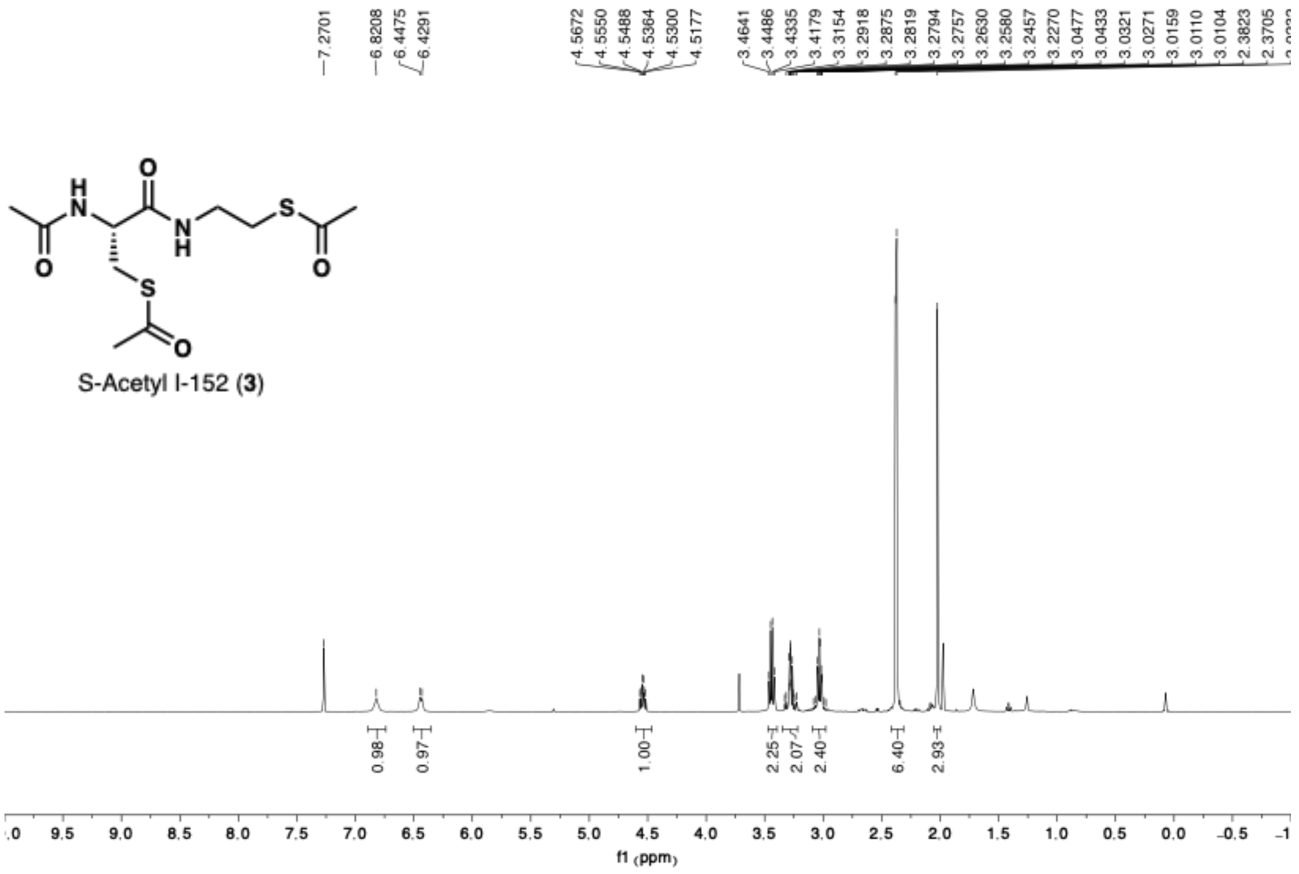

Figure S5. ^{1}H NMR spectrum (400 MHz) of S-Acetyl I-152 (**3**) in $CDCl_3$.

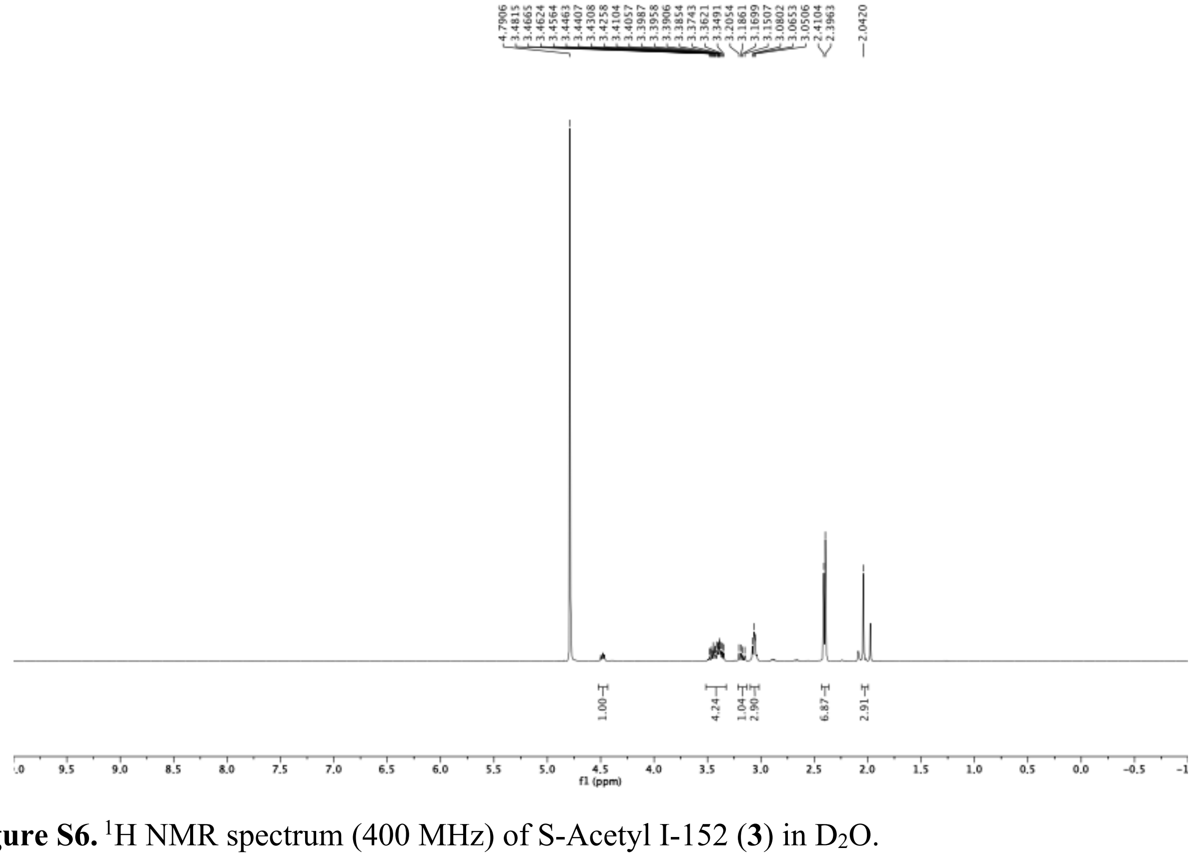

Figure S6. ^{1}H NMR spectrum (400 MHz) of S-Acetyl I-152 (**3**) in D_2O.

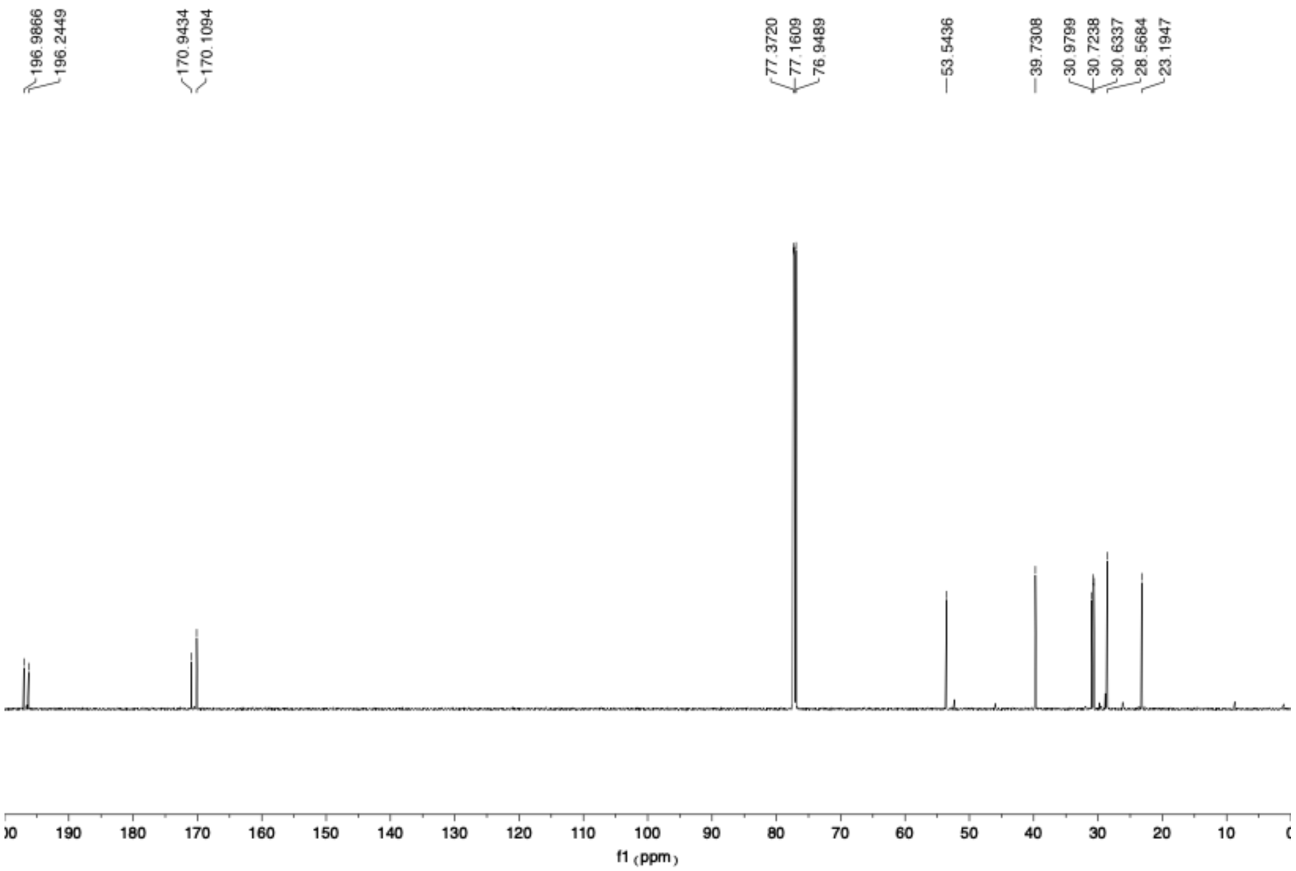

Figure S7. ^{13}C NMR spectrum (100 MHz) of S-Acetyl I-152 (**3**) in $CDCl_3$.

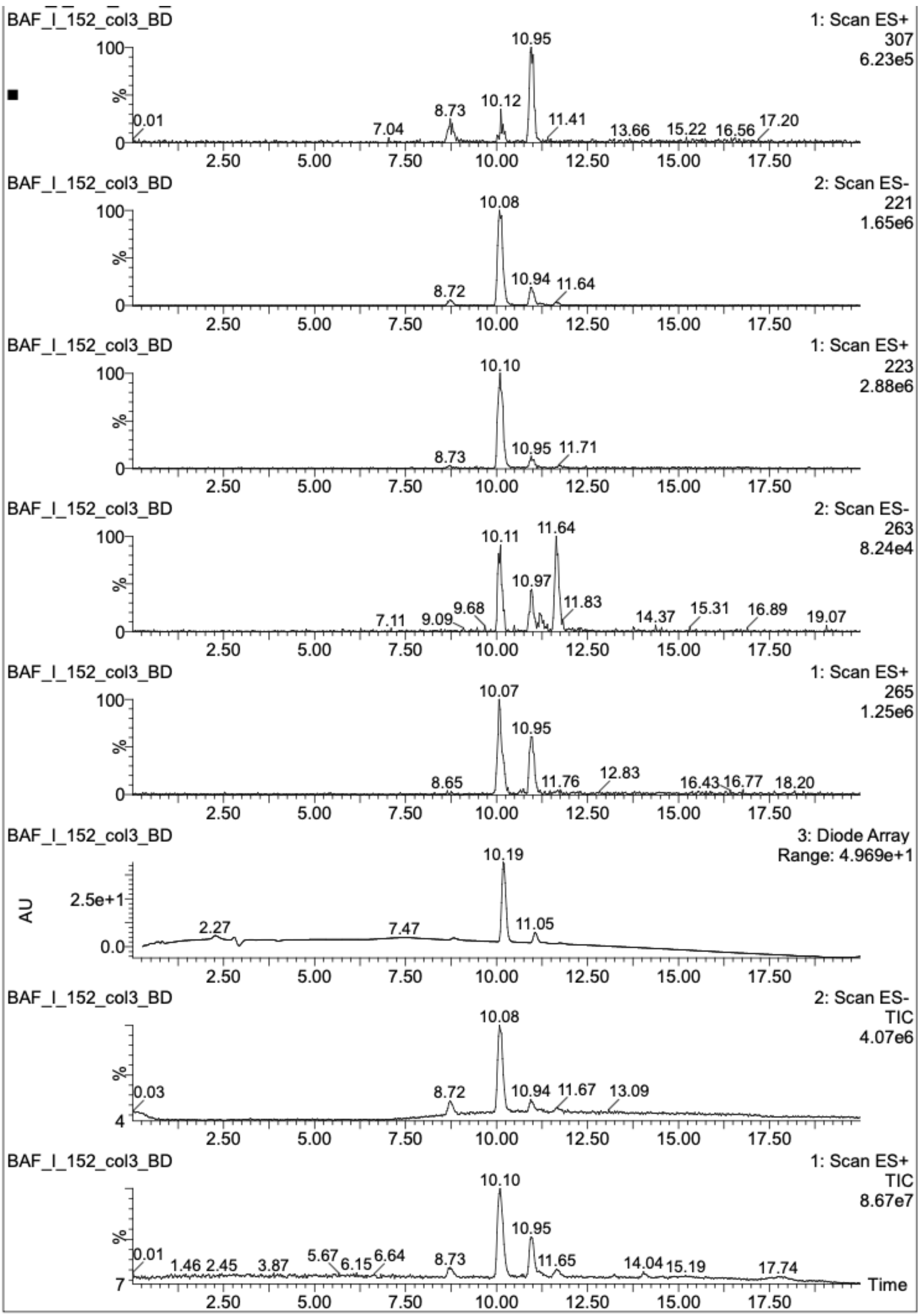

Figure S8. HPLC chromatogram of I-152 (**1**) a t = 0 s

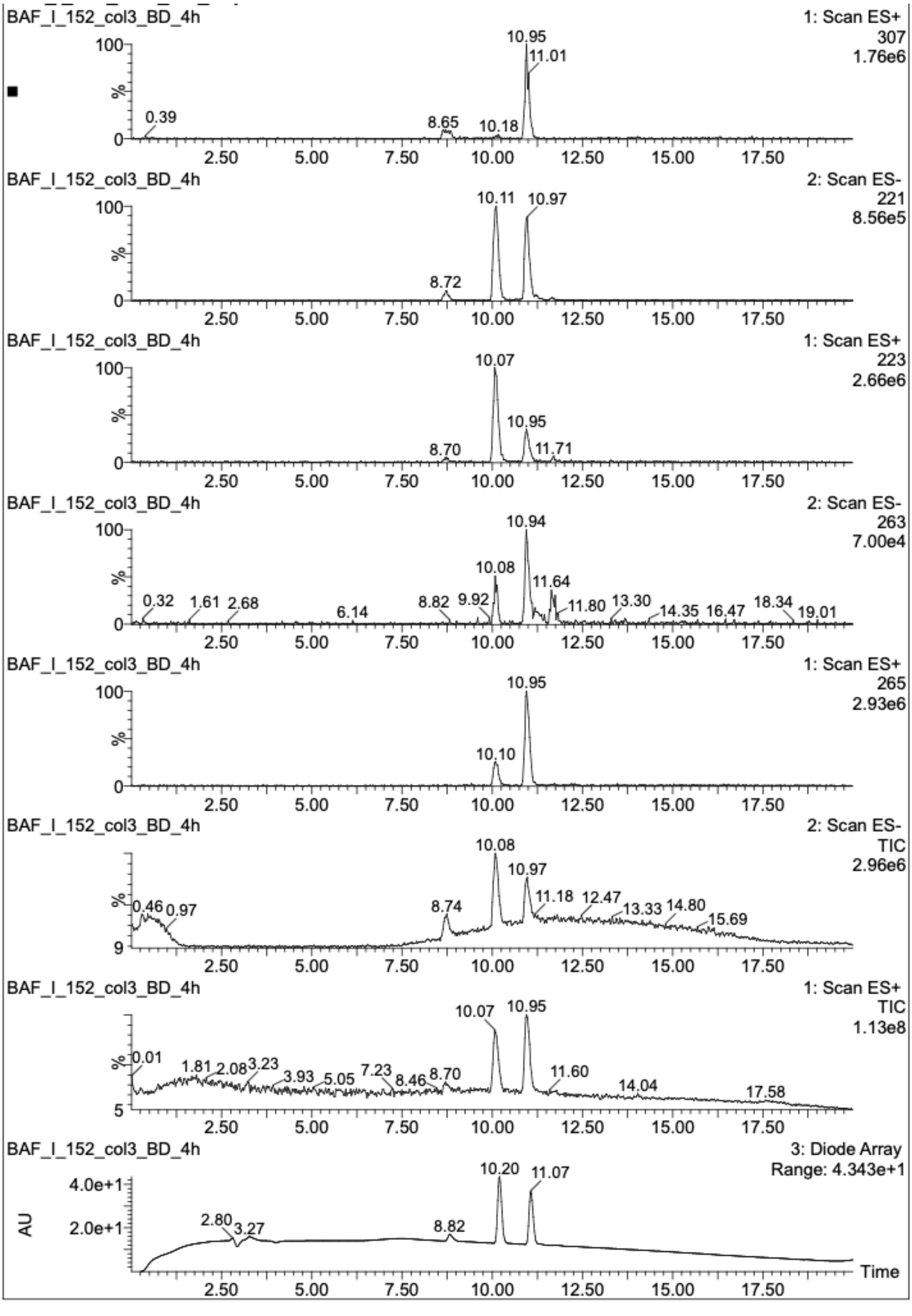

Figure S9. HPLC chromatogram of I-152 (**1**) a t = 4 h

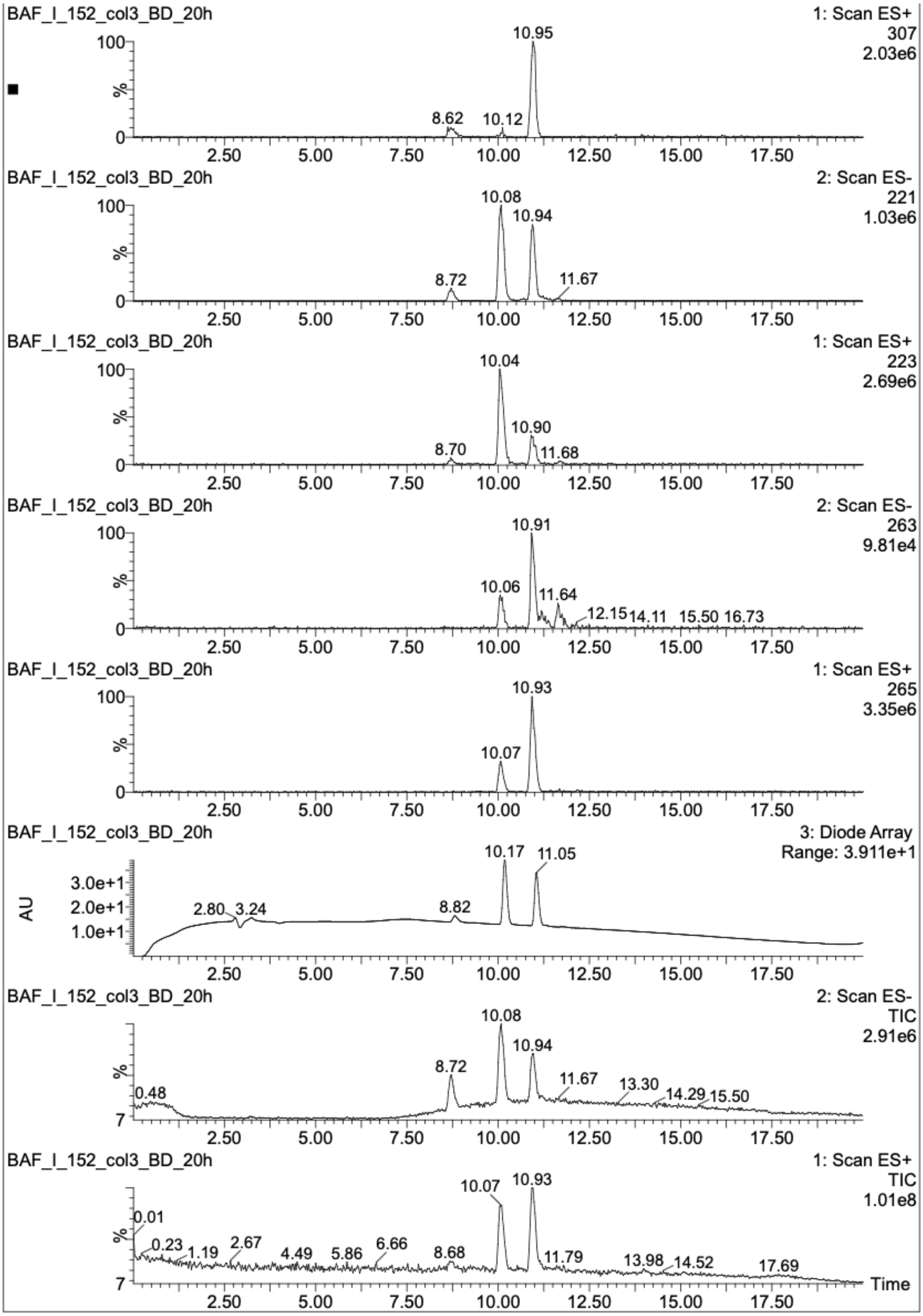

Figure S10. HPLC chromatogram of I-152 (**1**) a t = 20 h

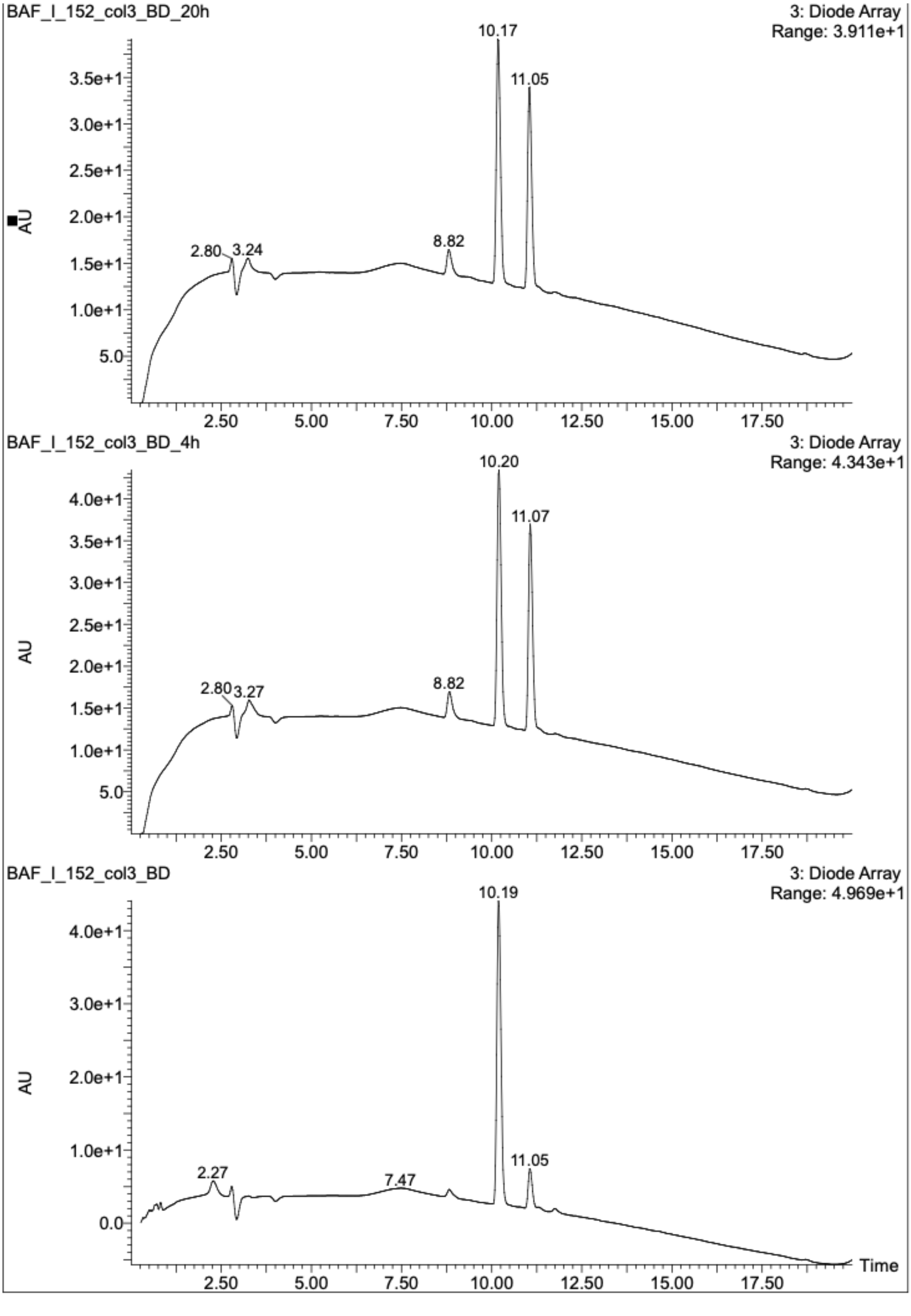

Figure S11. Comparison of HPLC chromatogram of I-152 (**1**) at t = 0 s, t = 4 h and t = 20 h.

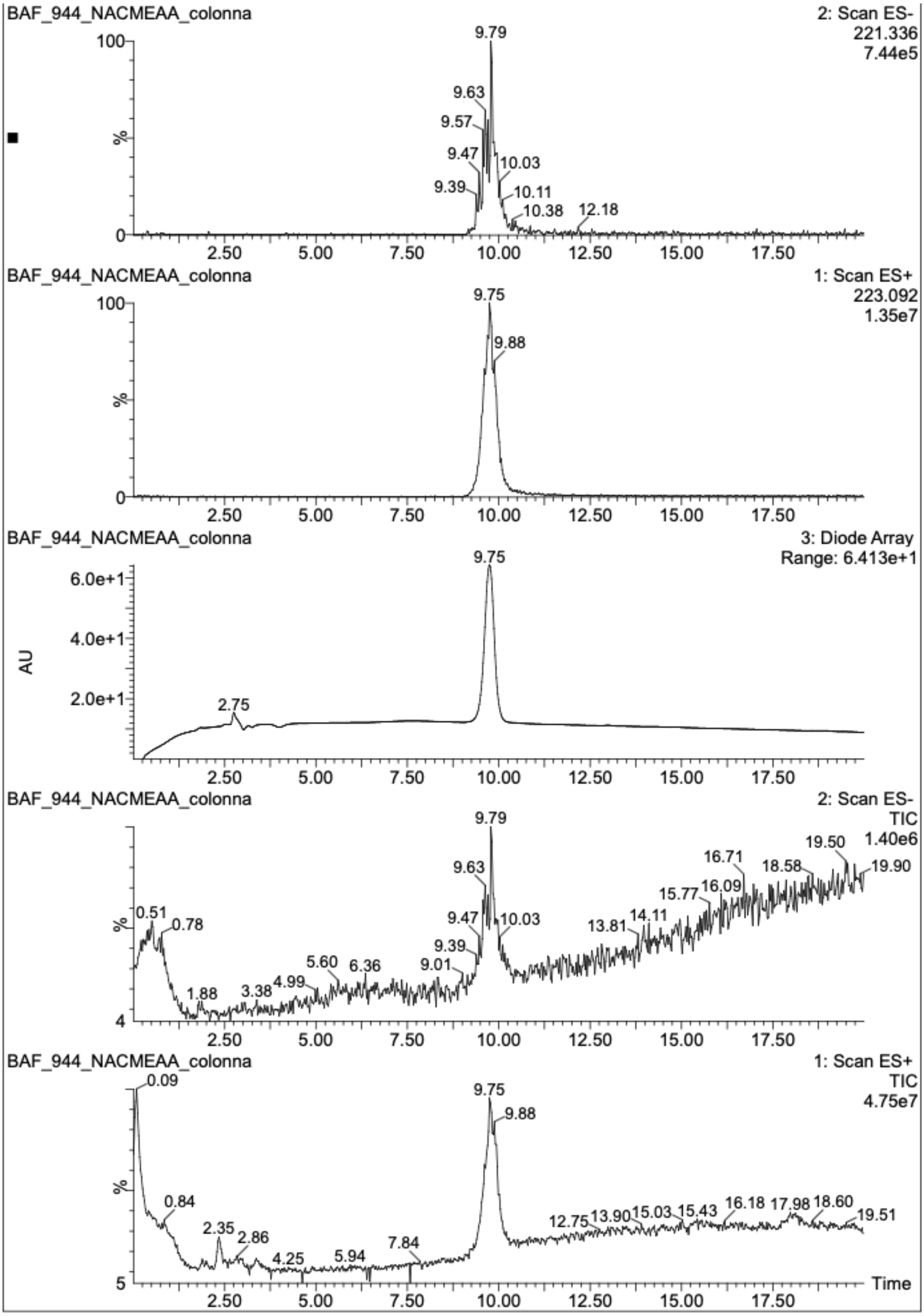

Figure S12. HPLC chromatogram of NACMEAA (**2**)

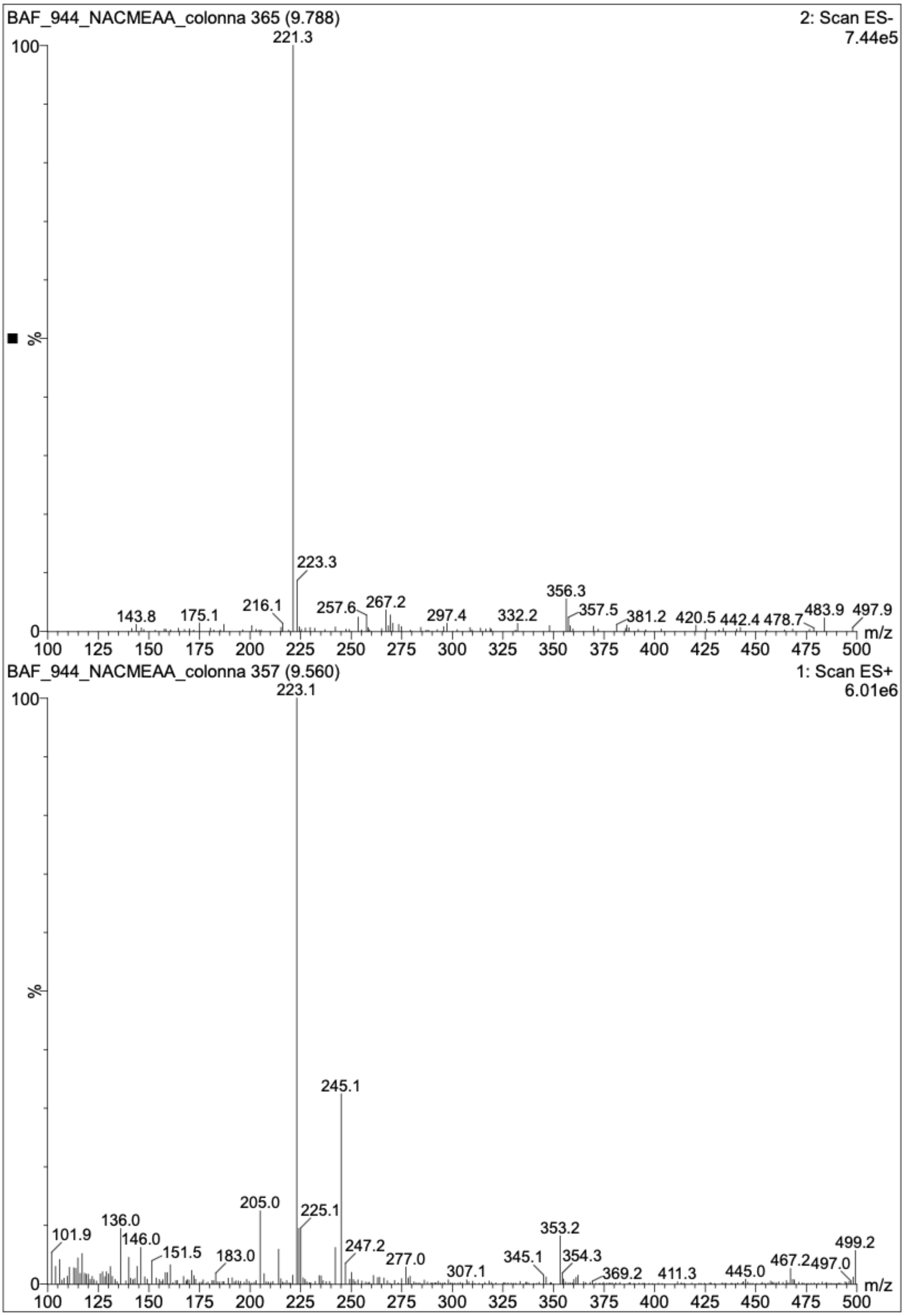

Figure S13. MS chromatogram of NACMEAA (**2**).

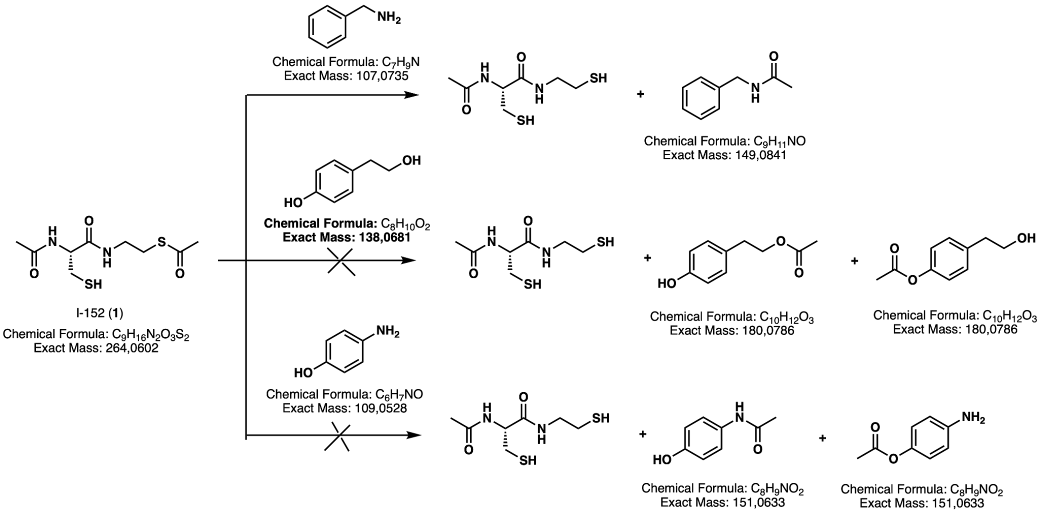

Scheme S1. Reaction of I-152 (**1**) with benzylamine, tyrosol and *p*-amino phenol.

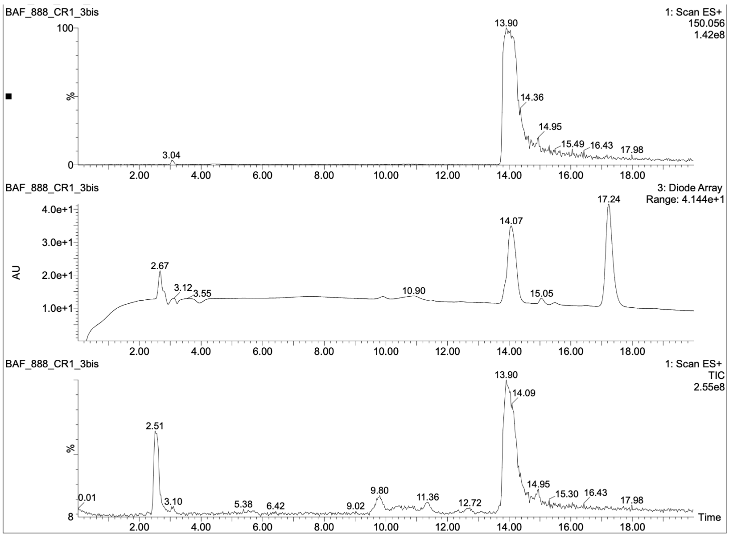

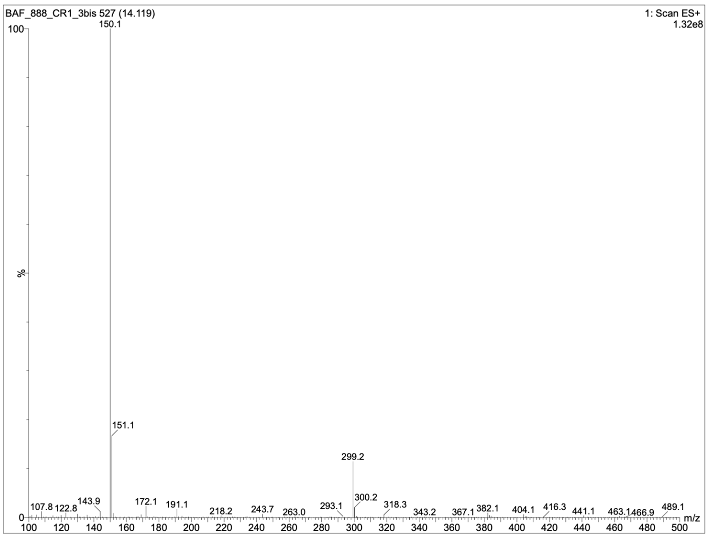

Figure S14. HPLC-MS chromatogram of the reaction between I-152 (**1**) and benzylamine

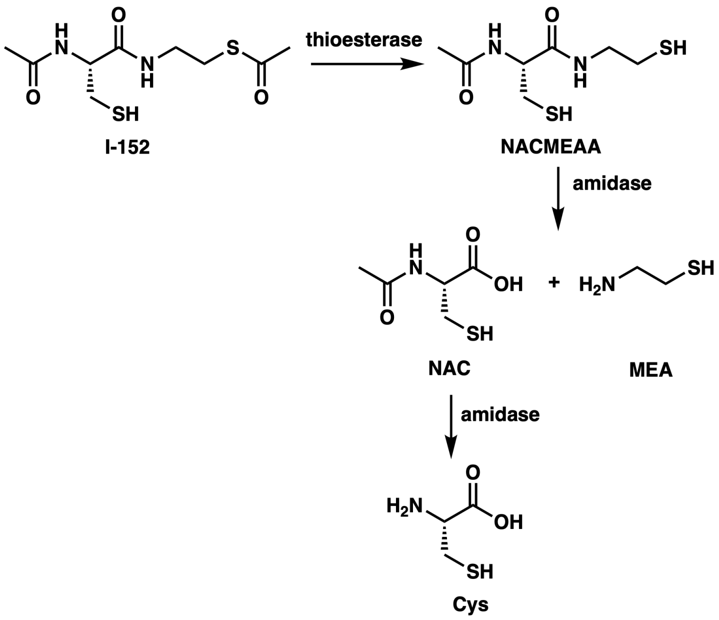

Scheme S2. Hydrolysis of I-152 (**3**)

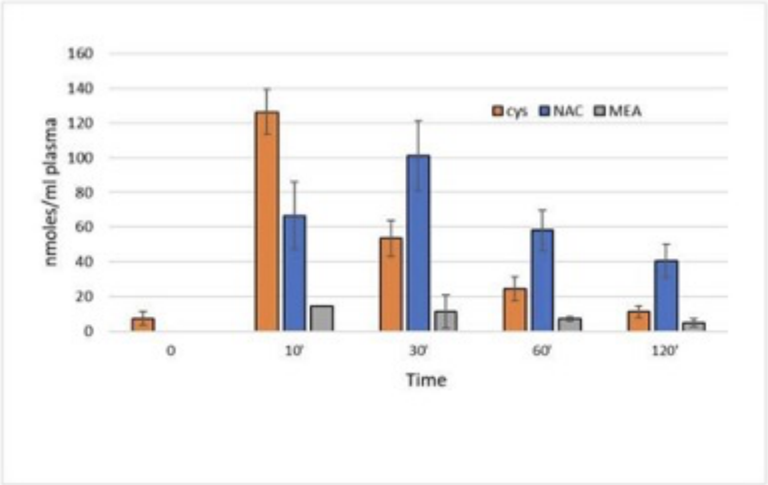

Figure S15. NAC, MEA and cys detection in human plasma incubated with I-152 (1 mM) at 37°C. The thiol species were determined through HPLC analysis according to the previously described procedure.[1] Briefly, at each time point 0.12 mL plasma were precipitated with a protein precipitating solution containing metaphosphoric acid. After centrifugation, 10% (v/v) DTNB; Ellman's Reagent, 5,5'-Dithiobis-(2-Nitrobenzoic Acid) was added to the supernatants for thiol determination. Quantification of thiol species was obtained by injection of standards of known concentrations, and the values are the mean ± S.D. of at least 7 independent experiments.

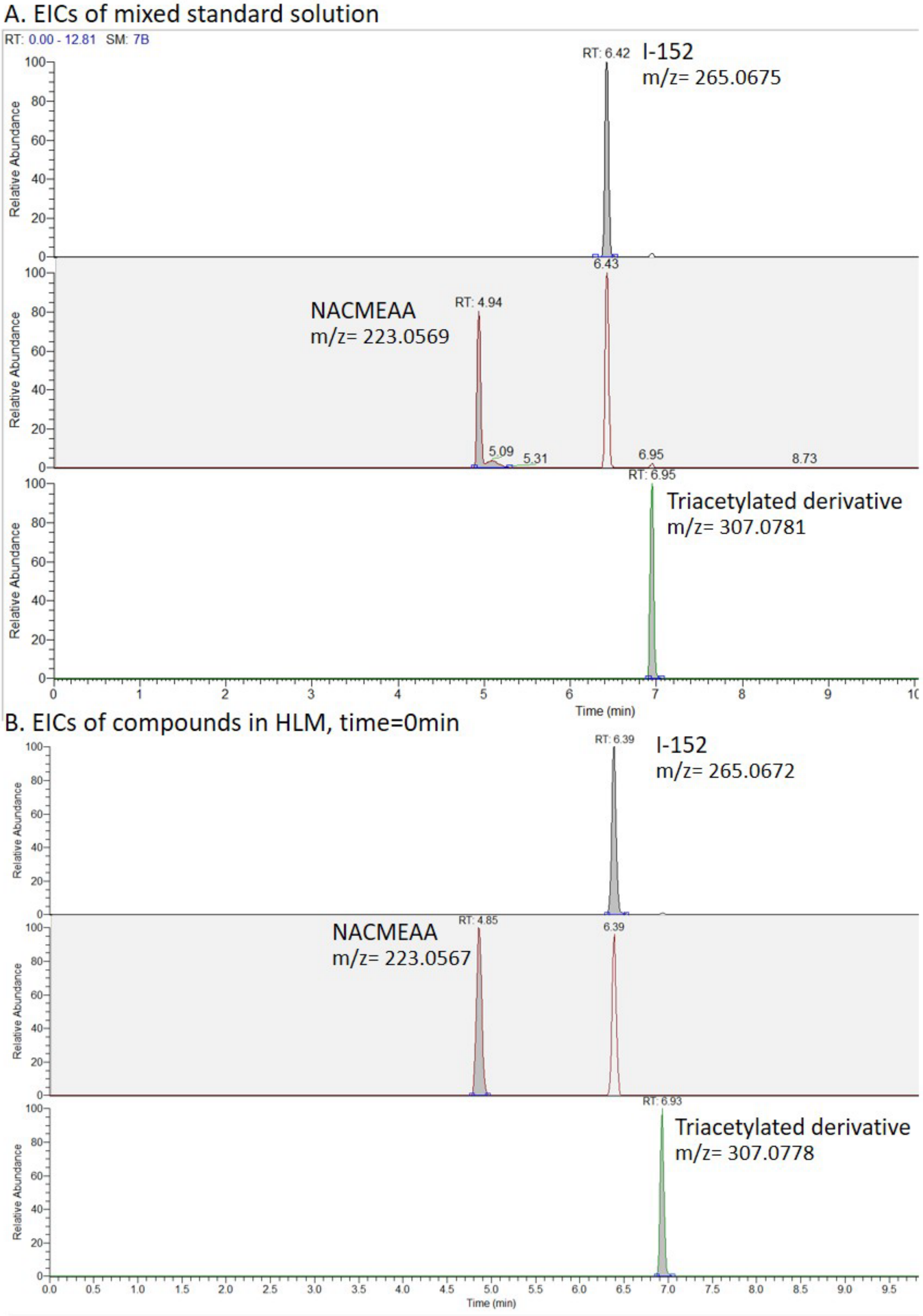

Figure S16. Representative overlaid extracted ion current (EIC) chromatograms of a mixed standard solution (A) and a sample of I-152 incubated in HLM at time 0 min (B).

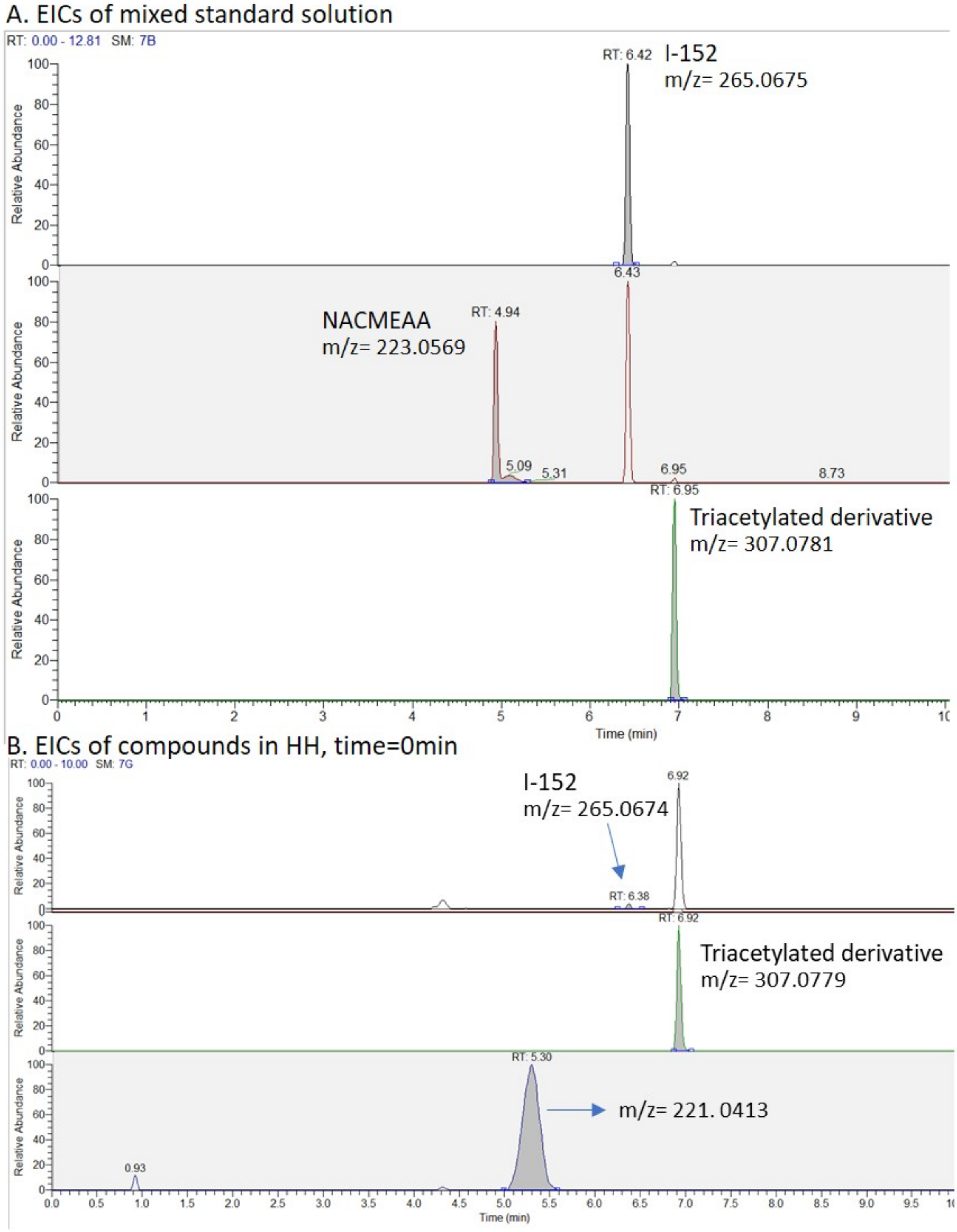

Figure S17. Representative overlaid extracted ion current (EIC) chromatograms of a mixed standard solution (A) and a sample of I-152 incubated in HH at time 0 min (B).

Compound	Compound	Concentration (µM)	% inhibition		
			n1	n2	mean
I-152 (**3**)	I-152 (**3**)	0.1	15.13	15.29	15.21
I-152 (**3**)	I-152 (**3**)	1	13.33	13.28	13.30
I-152 (**3**)	I-152 (**3**)	10	11.90	12.52	12.21
I-152 (**3**)	I-152 (**3**)	50	10.05	13.17	11.61
I-152 (**3**)	I-152 (**3**)	75	13.31	15.68	14.49
I-152 (**3**)	I-152 (**3**)	100	17.19	19.16	18.17
Time-Matched Vehicle Control	0.33% DMSO Addition 1		18.42	16.70	17.56
Positive References Control	Verapamil	0.03	22.80	22.68	22.74
Positive References Control	Verapamil	0.1	34.42	31.39	32.90
Positive References Control	Verapamil	0.3	37.26	36.78	37.02
Positive References Control	Verapamil	1	68.26	70.56	69.41
Positive References Control	Verapamil	3	83.66	83.72	83.69
Positive References Control	Verapamil	10	95.39	96.41	95.90

Compound	Mode	Estimated IC_{50} (µM)
I-152 (**3**)	Antagonist	<25%
Verapamil	Antagonist	0.343

Table S1. Effect of compound I-152 (**3**) on hERG assay. Concentration-dependent inhibition of hERG tail current measured using the Qube automated patch-clamp CiPA assay. Data represent mean percentage inhibition of hERG current at concentrations ranging from 0.1 to 100 µM. Verapamil was used as a positive control to validate assay performance.

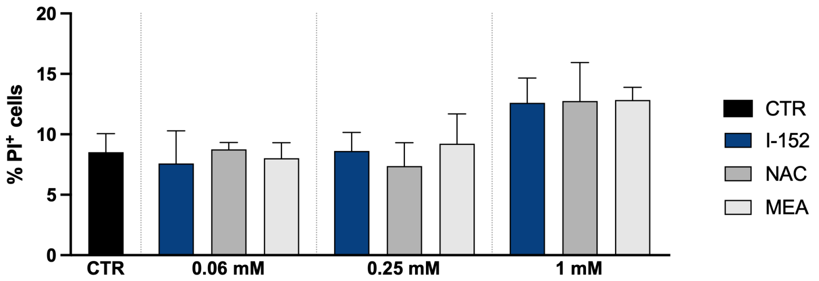

Figure S18. Comparative toxicity studies between I-152 and NAC or MEA. HEK293T cells were treated with increasing concentrations of I-152, NAC or MEA, and the cell viability was evaluated at 24 h by propidium iodide (PI) uptake using flow cytometry. Data are reported as % PI-positive cells. The data represent the mean ± SD of three biological replicates and two technical replicates.

References

(1) Crinelli, R.; Zara, C.; Galluzzi, L.; Buffi, G.; Ceccarini, C.; Smietana, M.; Mari, M.; Magnani, M.; Fraternale, A. Activation of NRF2 and ATF4 Signaling by the Pro-glutathione Molecule I-152, a Co-drug of *N*-Acetyl-cysteine and cysteamine. *Antioxidants* **2021**, *10*, 175.
